# A systematic literature review of frequency of vaso-occlusive crises in sickle cell disease

**DOI:** 10.1186/s13023-021-02096-6

**Published:** 2021-11-02

**Authors:** Ahmar U. Zaidi, Alexander K. Glaros, Soyon Lee, Taiji Wang, Rhea Bhojwani, Eric Morris, Breanne Donohue, Jincy Paulose, Şerban R. Iorga, Dave Nellesen

**Affiliations:** 1 Children’s Hospital of Michigan, Detroit, MI USA; 2 Novartis Pharmaceutical Corporation, East Hanover, NJ USA; 3 Analysis Group, Inc., 1010 El Camino Real, Suite 310, Menlo Park, CA 94025 USA; 4 Central Michigan University, Mount Pleasant, MI USA

**Keywords:** Vaso-occlusive crisis, Pain crisis, Sickle cell disease, Real-world, Incidence, Prevalence

## Abstract

**Background and purpose:**

Sickle cell disease (SCD) is a collection of rare inherited blood disorders affecting approximately 100,000 people in the U.S. and 20–25 million people globally. Individuals with SCD experience recurrent episodes of severe and unpredictable pain that are caused by vaso-occlusive crises (VOCs), a hallmark of the disease. VOCs are the primary cause of hospitalization in SCD, result in missed workdays and school days, and decrease quality of life (QoL). Although VOCs cause significant burden in the lives of individuals with SCD, there is no synthesis on the frequency of VOCs in the real world. This systematic literature review sought to identify literature describing the frequency of VOCs experienced by individuals with SCD in real-world settings.

**Methods:**

MEDLINE and 6 congresses were searched (date range: January 1, 2000 to June 30, 2020). Studies were reviewed independently by two researchers. Studies assessing frequency or prevalence of VOCs or VOC-related outcomes were included.

**Results:**

Of 1438 studies identified in the search, 52 met pre-specified inclusion and exclusion criteria. Reported frequency of VOCs varied widely ranging from a mean or median of 0 VOCs/year to 18.2 VOCs/year. The proportion of patients experiencing ≥ 3 VOCs/year ranged from 4 to 67% and the proportion of patients experiencing ≥ 5 VOCs/year ranged from 18 to 59%. Measures of VOC severity were limited, with 13 studies considering frequency of complicated VOCs and only 1 study reporting duration of VOC episodes.

**Conclusions:**

This is the first study to systematically assess published evidence pertaining to VOCs in real-world settings. Reported VOC frequency in real-world settings varied widely, with a majority of studies only considering VOCs managed in an inpatient or outpatient setting. Studies that considered VOCs managed at home reported a higher frequency of VOCs, suggesting that many studies may underestimate the frequency of VOCs. This systematic literature review (SLR) highlights the need for consistent reporting of (1) self-reported VOCs, including those managed at home, (2) definitions of VOCs, (3) complicated VOCs, and (4) duration of VOC episodes in literature.

**Supplementary Information:**

The online version contains supplementary material available at 10.1186/s13023-021-02096-6.

## Introduction

Sickle cell disease (SCD) is a collection of rare inherited blood disorders resulting from a mutation of hemoglobin that causes an abnormal sickling of red blood cells [1]. Approximately 100,000 people in the U.S. and 20–25 million people globally have SCD [2, 3].

Individuals with SCD have a reduced life expectancy and experience both acute and chronic complications. Sickled red blood cells, monocytes, platelets, and neutrophils tend to stick together, forming multicellular adhesion clusters in the bloodstream [4, 5]. These clusters impede regular blood flow and oxygenation, which can damage blood vessels and surrounding tissues via ischemia reperfusion injury, leading to a number of acute and chronic complications [1, 6, 7]. A clinical hallmark of SCD is vaso-occlusive crisis (VOC), often characterized by the sudden onset of severe pain [8]. Though there are frequent and common sites of pain in patients with SCD, this pain can occur in virtually any part of the body, often with multiple locations affected simultaneously [7, 9]. VOCs are the primary cause of hospitalizations for patients with SCD and are associated with increased mortality and organ damage [6, 10]. However, due to barriers to accessing care, a large proportion (51–79%) of patients manage their VOCs at home [11, 12]. Increased frequency of VOCs is associated with a significant decrease in health-related quality of life (HRQoL) and is also associated with greater absenteeism and overall productivity loss [13, 14]. In addition, VOCs are also associated with the development of other complications including acute chest syndrome (ACS), priapism, and hepatic/splenic sequestration, which decrease HRQoL and require extensive healthcare resource utilization [6, 10].

The objective of this systematic literature review (SLR) is to summarize published literature describing the frequency of study-defined VOCs experienced by pediatric and adult patients with SCD as reported in real-world settings globally. Clinical trial data were not included as trial populations may not be representative of the patient population encountered in the real-world setting. Inclusion and exclusion criteria can modify the frequency of VOCs experienced in a trial. Therefore, real-world populations are more inclusive and more accurately represent the burden of VOCs. The following research questions were assessed in the real-world setting: (1) How are VOCs defined? (2) What is the annual frequency of VOCs? (3) What is the duration of a VOC? (4) How often are VOCs managed in different care settings?

## Methods

### Literature search and screen

MEDLINE, including MEDLINE In-Process, database was searched via Ovid (date range: January 1, 2000 to June 30, 2020). Additional manual searches of 6 relevant and publicly available congress abstract databases were conducted to capture recent research (last 2 years) which may not yet be indexed in MEDLINE. Complete search strategies are provided in Additional file 1.

All studies identified in the literature search were reviewed independently and in parallel by two researchers using an Excel-based screening platform. Any disagreements were resolved by discussion between researchers or by independent arbitration by a third researcher. Studies were first screened by title and abstract. The full-texts of studies that met inclusion criteria were then reviewed using the same inclusion and exclusion criteria. Studies were included if frequency or prevalence of VOCs or VOC-related outcomes were reported. VOC data reported at study baseline or in the absence of treatment was extracted in cases where multiple values for frequency or prevalence of VOCs or VOC-related outcomes were reported. No restrictions were placed on VOC definition; VOC definitions were extracted as reported in the study and included as available.

Evidence synthesis of frequency of VOCs was based on the number of VOCs experienced by the population during a specific time period. Evidence synthesis of prevalence of VOCs was based on the proportion of the population that experiences at least 1 VOC at or during a specific time period. The time period over which VOCs were reported varied, we converted to annual frequency rates. Evidence synthesis of frequency of complicated VOCs included VOCs with concomitant ACS, priapism, and hepatic/splenic sequestration. Due to statistical and methodologic heterogeneity among studies, we did not carry out statistical pooling.

Clinical trials and retrospective analyses of clinical trials were excluded. Inclusion and exclusion criteria for both database and additional supplementary manual searches of congresses are provided in Additional file 2.

### Data extraction

Predefined variables were extracted from eligible studies by a single researcher and checked for accuracy by a second researcher. Extracted data included publication details, study methodology (e.g., design, number of patients), patient characteristics (e.g., age, race/ethnicity), and clinical outcomes (e.g., definition of VOC, annual frequency of VOCs, frequency of complicated VOCs).

Studies that reported timing of VOCs or VOC-related outcomes were extracted and included in the data analysis. Frequency of VOCs was extracted for the time-period reported in the study; the frequency was also annualized for comparability purposes between studies. Studies that did not report timing of VOCs or VOC-related outcomes were not extracted nor included in the data analysis and were tabulated in Additional file 3.

### Quality assessment

Study quality assessment was conducted on all extracted studies using the International Society of Pharmacoeconomics and Outcomes Research (ISPOR) questionnaire for prospective and retrospective observational studies [15]. Studies were assessed by a single researcher and checked for accuracy by a second researcher. The questionnaire has 12 questions, of which, 4 related to study “relevance” and 8 related to study “credibility,” which can be answered as “Y” (Yes), “N” (No), “NR” (Not Reported), and “NA” (Not Applicable). The total number of affirmative responses to quality elements were tabulated for each study as the primary measure of study quality. In addition, the number of affirmative responses for study relevance-related and study credibility-related questions were separately tabulated for each study.

## Results

### Study selection

**Fig. 1:**
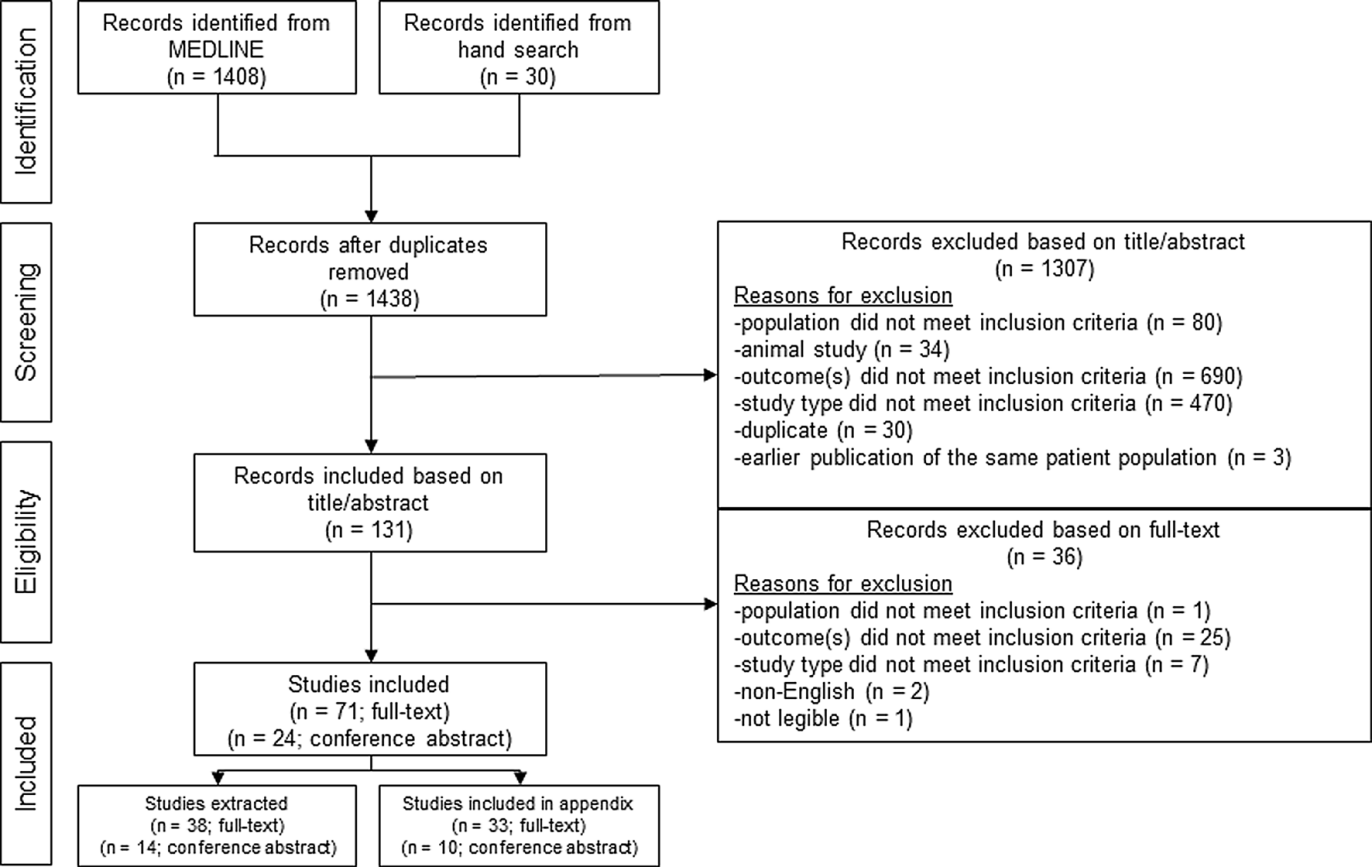
PRISMA flow diagram of study inclusions and exclusions

The initial search for studies yielded 1438 studies, of which 1408 were from an electronic database (date range: January 1, 2000 to June 30, 2020) and 30 were from manual searches of recent congresses (last 2 years). Title abstract screening excluded 1307 studies (outcome did not meet inclusion criteria [n = 690]; study type did not meet inclusion criteria [n = 470], population did not meet inclusion criteria [n = 80]; animal study [n = 34]; duplicate [n = 30]; earlier publication of the same patient population [n = 3]), leaving 131 full-text articles to be screened for final inclusion in the SLR. Of those, 36 were excluded after full-text screening (outcome did not meet inclusion criteria [n = 25]; study type did not meet inclusion criteria [n = 7]; non-English [n = 2]; population did not meet inclusion criteria [n = 1]; not legible [n = 1]) leaving a final set of 95 for inclusion in the SLR (Fig. 1). Of the 95 studies included in the SLR, 52 studies reported timing of VOCs and VOC-related outcomes were extracted and included in the data analysis. A full list of included publications is provided in Additional file 4.

Figure 1 shows a PRISMA flow diagram summarizing the literature search process and exclusion counts by pre-specified inclusion/exclusion criteria.

### Study characteristics

The analysis was based on 52 studies, representing a mix of retrospective observational (23 studies), prospective observational (21 studies), and cross-sectional (8 studies). This includes studies from the U.S. (19), Asia (10), Europe (9), Caribbean/South American (6), and Africa (3); as well as 5 studies comprising multiple countries.

Sample sizes varied widely across the 52 studies from a minimum of 15 patients to a maximum of 44,033. The mean sample size was 2794, median was 254, and the standard deviation was 7923. Of the 6 studies with sample sizes larger than 5000, 4 were analyses of administrative healthcare claims and 2 were analyses of electronic health records.

Patient demographics included a mix between pediatric-only (< 18 years; 15 studies), adult-only (≥ 18 years; 14 studies), and mixed pediatric and adult (23 studies).

The availability of treatment information varied widely across the 52 studies. More than a third of studies did not report type of treatment received for SCD (18 studies). Among the studies that did report type of treatment received for SCD, 12 studies reported hydroxyurea use only (proportion of patients treated with hydroxyurea ranged from 2 to 100% among these studies), 12 studies reported multiple treatments including hydroxyurea, and 10 studies reported some other form of treatment information. Additional treatment information can be found in Additional file 5.

### Clinical outcomes

Thirty-nine studies reported VOC frequency or prevalence outcomes, of which 33 reported frequency of VOC and 25 reported prevalence of VOC. Among studies that reported prevalence of VOC, 11 reported the prevalence distribution.

Overall there is variability in the definition of VOC in literature. Among the 39 studies that reported VOC frequency or prevalence outcomes, the *variable* VOC definition in 15 studies required a healthcare visit (outpatient [OP], inpatient [IP], and/or emergency department [ED]); in 11, a hospitalization event; in 4, a healthcare visit documented by a medical claim; and in 5, considered self-reported VOCs, including VOCs that were managed at home. Four studies did not report how VOCs were defined within the study. A full list of definitions of VOCs, as reported by the original study authors, is provided in Additional file 4.

**Fig. 2:**
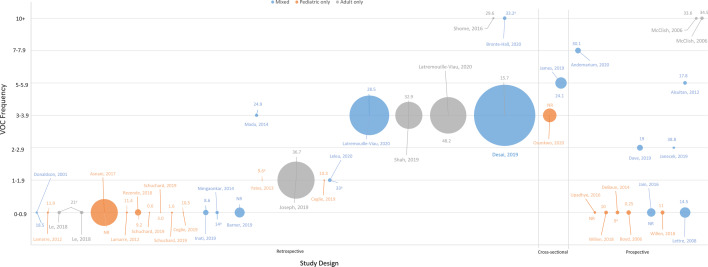
Reported frequency of VOCs by study design, patient age group, and sample size. The vertical axis represents the reported frequency of VOCs in the real world. The horizontal axis represents the study design type. The color of the circular data point represents the age group of the patient population. The size of the circular data point represents the patient population size. Median age is reported where indicated (^a^), otherwise mean age is reported

Reported mean or median VOC frequency varied widely in literature with a minimum of 0 VOCs/year [16] to a maximum of 18.2 VOCs/year [17]. Studies reporting higher frequencies of VOCs include a mixture of pediatric and adult patient populations, study countries, patient population size, and study type, as depicted in Fig. 2. The SLR identified 2 different patient cohorts across 4 publications which considered self-reported VOCs [18–21]. The first was the Sickle Cell World Assessment Survey (SWAY) of patients with SCD (≥ 16 years) from multiple countries across North and South America, Europe, and Africa [18, 19]. SWAY defined VOCs as severe patient-reported pain crises and reported a mean of 5.2 VOCs/year and a median of 3 VOCs/year. Among the 384 U.S. patients, a mean of 7.1 VOCs/year was reported [20]. The second was a prospective cohort study of 226 adult U.S. patients with SCD who completed daily pain dairies for up to 6 months [21]. In this study, VOCs were defined as consecutive days in which a patient reports pain, and means of 6.0 VOCs/6 months and 6.8 VOCs/6 months were reported among male and female patients, respectively. Key study characteristics of all studies that reported VOC frequency, ranked from lowest to highest reported VOC frequency, are provided in Table 1.

**Table 1:** VOC frequency studies

Study identifier	Country	N	Mean age	VOC definition	# of reported VOCs/year			
					Mean	SD	Range	IQR
Le et al. [16]	Belgium	139	21^a^	Hospitalization	0/100 PY^a,b^	‒	‒	‒
					29.6/100 PY^a,c^			
Donaldson et al. [22]	Jamaica	50	18.5	NR	5.63/100 PY^d^	‒	‒	‒
Upadhye et al. [23]	India	75	NR	NR	9.7/100 PY	‒	‒	‒
Lamarre et al. [24]	French West Indies	49	11.9	Hospitalization	0.26^e^	0.39	‒	‒
		44	11.4		0.41^f^			
Willen et al. [25]	Multiple	165	10	Hospitalization	0.27^a^	‒	‒	‒
Asnani et al. [26]	Jamaica	8504	NR	Healthcare visit (OP or IP) to center; recorded in database	0.4	0.7	‒	‒
DeBaun et al. [27]	U.S	159	9^a^	Hospitalization and treatment with opioids	0.46^a^	‒	‒	‒
Boyd et al. [28]	U.S	242	0.25	Healthcare visit (OP or IP) to clinic	0.47^g^	‒	‒	‒
Rezende et al. [29]	Brazil	461	9.2	Healthcare visit (OP or IP) to center	51/100 PY	‒	48.9–53.4	‒
Schuchard et al. [30]	U.S	35	0.6	Healthcare visit (OP or IP) to center	0.15^h^	‒	‒	‒
		13	1.6		0.9^i^			
		17	3.0		0.52^j^			
Ceglie et al. [31]	Italy	16	10.5	Hospitalization at study center	0.6^k^	‒	‒	‒
		23	10.3		1.6^l^			
Inati et al. [32]	Lebanon	335	8.6	Hospitalization	0.6	0.6	0.04–2.4	‒
Jain et al. [33]	India	833	NR	Healthcare visit (OP or IP) to center	64/100 PY	‒	‒	‒
Lettre et al. [34]	Multiple	1275	14.5	NR	0.7/PY	1.4	‒	‒
Nimgaonkar et al. [35]	India	137	14^a^	NR	0.7	‒	‒	‒
Barner et al. [36]	U.S	1190	NR	Healthcare visit (OP, IP, or ED)	4.1/5 year study period	5	‒	‒
Willen et al. [37]	U.S	197	11	Hospitalization and treatment with opioids	0.86	‒	‒	‒
Yates et al. [38]	Multiple	15	9.6^a^	Hospitalization	1.18^a,m^	‒	0–1.94	‒
Joseph et al. [39]	U.S	16,092	36.7	Healthcare visit (OP, IP, or ED); Texas Medicaid claims	1.2	‒	‒	‒
Leleu et al. [40]	France	157	33^a^	Healthcare visit (OP, IP, or ED); French National Health Data System	1.9^n^	‒	‒	‒
Janecek et al. [41]	U.S	66	38.8	Healthcare visit (OP, IP, or ED)	2^o^	‒	‒	‒
Dave et al. [42]	India	404	19	Healthcare visit (OP or IP) to care system	2.77	‒	‒	‒
Madu et al. [43]	Nigeria	126	24.9	Healthcare visit (OP or IP) to center	3	4.8	‒	‒
Osunkwo et al. [18]	Multiple	2145	NR	Self-reported (patient survey; hospitalization, ED, managed at home)	3^a^	‒	‒	2–6
Latremouille-Viau et al. [44]	U.S	18,287	28.5	Healthcare visit (OP, IP, or ED); Medicaid and Medicare claims	3.1^p^		‒	‒
		15,431	48.2		3.4^q^			
Shah et al. [45]	U.S	8521	32.9	Healthcare visit (OP, IP, or ED); Medicaid claims	3.31	‒	‒	‒
Desai et al. [46]	U.S	44,033	15.7	Healthcare visit (≥ 2 OP, or ≥ 1 IP, or ED); Medicaid claims	3.71	‒	‒	‒
James et al. [19]	Multiple	1513	24.1	Self-reported (patient survey; hospitalization, ED, managed at home)	5.2	‒	‒	‒
Alsultan et al. [47]	Saudi Arabia	159	17.8	Hospitalization for > 48 h	5.6	5.7	0–36	‒
Andemariam et al. [20]	U.S	384	30.1	Self-reported (patient survey; hospitalization, ED, managed at home)	7.1	5.7	‒	‒
Bronte-Hall et al. [48]	U.S	149	33.2^a^	Healthcare visit (OP or IP) to center	10.4	‒	‒	‒
McClish et al. [21]	U.S	87	33.6	Self-reported (patient diary)	6.0/6 months^k^	‒	‒	‒
		139	34.5		6.8/6 months^l^			
Shome et al. [17]	Bahrain	51	29.6	Healthcare visit (OP or IP) to center	18.2^m^	17.5	‒	‒

*ED* emergency department, *IP* inpatient, *IQR* interquartile range, *NR* not reported, *OP* outpatient, *PY* patient-year, *SCD* sickle cell disease, *SD* standard deviation, *VOC* vaso-occlusive crisis

^a^Median reported

^b^Adults study arm

^c^Adolescents study arm

^d^Cases arm, patients with steady-state HBF levels below 1%

^e^HbSC study arm

^f^HbSS study arm

^g^Non-asthmatic study arm

^h^Patients who started hydroxyurea between 5 months and 1 year of age

^i^Patients who started hydroxyurea between 1 and 2 years of age

^j^Patients who started hydroxyurea between 2 and 5 years of age

^k^Male study arm

^l^Female study arm

^m^Pre-hydroxyurea therapy study arm

^n^Patients who had at least 2 inpatient stays for SCD diagnosis/chronic long-term SCD, or a reimbursement for Siklos/Hydrea

^o^Pre-assessment study arm

^p^Medicaid claims study arm

^q^Medicare claims study arm

Prevalence of VOCs were reported in 10 studies, 4–67% of patients experienced ≥ 3 VOCs/year and 18.2–59% of patients experienced ≥ 5 VOCs/year. The 3 studies that considered self-reported VOCs were the upper range for both proportion of patients experiencing ≥ 3 VOCs/year and ≥ 5 VOCs/year [13, 19, 20]. However, 2 studies which considered VOCs managed in an OP, IP, or ED setting, but did not consider VOCs managed at home, reported a substantial proportion of patients (9.2–15.8%) who experienced a high number of VOCs (> 10 VOCs/year) [45, 49]. Key study characteristics of all studies that reported a VOC prevalence distribution are provided in Table 2. Only one study reported the duration of VOC episodes [39]. The analysis of claims data (N = 16,092) from the Truven Health MarketScan® Commercial Claims and Encounters Database (2000–2018) reported the mean duration of VOC episodes varied by treatment setting: inpatient (11.7 days), emergency room (2.3 days), and outpatient (1.9 days).

**Table 2:** VOC prevalence distribution studies

Study identifier	Country	N	Mean age	VOC definition	Distribution of VOC, %
Adekile et al. [ 53]	Kuwait	396	19.2	Hospitalization	0/year: 3.3
					1/year: 54.8
					2–3/year: 13.2
					> 3/year: 18.9
Aloni et al. [54]	Democratic Republic of Congo	168	10	Hospitalization > 48 h	0/year: 3.6
					1–3/year: 51.8
					> 3/year: 44.6
Andemariam et al. [20]	U.S	384	30.1	Self-reported (patient survey; hospitalization, ED, managed at home)	0/year: 4
					1–4/year: 38
					≥ 5/year: 59
Bailey et al. [14]	England	15,076	30^a^	Healthcare visit (OP, IP, or ED)	≥ 3/year: 4
Dave et al. [47]	India	404	19	Healthcare visit (OP or IP) to care system	0/year: 11.9
					1/year: 15.6
					2/year: 22.8
					≥ 3/year: 49.8
Delicou et al. [23]	Greece	254	NR	Healthcare visit (OP, IP, or ED)	0/year: 25.3
					1–5/year: 50.6
					5–10/year: 8.3
					> 10/year: 15.8
James et al. [19]	Multiple	1513	24.1	Self-reported (patient survey; hospitalization, ED, managed at home)	0/year: 8
					1–4/year: 51
					≥ 5/year: 40
Latremouille-Viau et al. [49]	U.S	18,287^b^	28.5	Healthcare visit (OP, IP, or ED); Medicaid and Medicare claims	0/year: 36.1
					1/year: 17
					≥ 2/year: 46.8
		15,431^c^	48.2		0/year: 44.9
					1/year: 11.4
					≥ 2/year: 43.7
Rizio et al. [13]	U.S	303	34.4	Self-reported (ASCQ-Me)	0/year: 8.9
					1/year: 9.6
					2/year: 14.5
					3/year: 19.8
					≥ 4/year: 47.2
Shah et al. [22]	U.S	8521	32.9	Healthcare visit (OP, IP, or ED); Medicaid claims	0/year: 52.3
					1/year: 14.7
					2/year: 6.7
					3/year: 4.6
					4/year: 3.7
					5/year: 2.7
					6/year: 2
					7/year: 1.5
					8/year: 1.4
					9/year: 1.4
					> 10/year: 9.2
van Tuijn et al. [55]	Netherlands	95	37^a^	Hospitalization	0/year: 23
					0–1/year: 43
					> 1/year: 16

*ASCQ-Me* adult sickle cell quality of life measurement information system, *ED* emergency department, *IP* inpatient, *NR* not reported, *OP* outpatient, *VOC* vaso-occlusive crisis

^a^Median reported

^b^Medicaid claims study arm

^c^Medicare claims study arm

### Complicated VOCs

Complicated VOCs include ACS, priapism, and hepatic/splenic sequestration. Only 13 unique studies reported frequency of complicated VOC: ACS (11 studies), hepatic/splenic sequestration (4 studies). No studies reported the frequency of priapism. Frequency of complicated VOC was < 1/year.

## Quality assessment metrics

Study quality was assessed for all 52 extracted studies. The number of affirmative responses to quality elements were tabulated for each study to determine study quality. Among the assessed studies, the mean number of affirmative responses to quality elements was 7.25 (median = 7), out of a total possible score of 12. Fourteen of the 52 extracted studies were conference abstracts which provided less information and a lower mean average score 6.2 (median = 6), out of a total possible score of 12. All studies had a similar high scoring for study relevance (2–3, out of a total possible score of 4) but there was more variability in scoring credibility (2–6, out of a total possible score of 8). No association was detected between the quality scoring of a study and rate of reported VOCs (correlation coefficient = 0.2).

## Discussion

The aim of this SLR was to assess published studies reporting the frequency of VOCs experienced by pediatric and adult patients with SCD as reported in real-word settings. Real-world studies have more inclusive patient populations and the potential to more accurately and completely capture the burden of VOCs and represent wider populations.

Definitions of VOCs, where present, differed across real-world studies with few commonalities except the inclusion of “pain” or “crisis”. There was also no consensus in the specificity of definition (e.g., a “composite of pain” versus “pain in the extremities, back, abdomen, chest, or head for which no explanation other than SCA could be found, lasting at least 2 h, leading to a clinic visit, and which was not classified as one of the following: skeletal/joint events, ACS, right upper quadrant pain, dactylitis, neurologic events, anemic episodes, febrile illness, and priapism”). Furthermore, explicit exclusions of certain symptoms within some definitions (e.g., “headaches”, “pain […] which was not classified as one of the following: skeletal/joint events, ACS, right upper quadrant pain, dactylitis, neurologic events, anemic episodes, febrile illness, and priapism”) further complicate the conceptual framework for VOC.

Operationalization of VOCs differed as well. Reported mean or median VOC frequency varied widely in literature with a minimum of 0 VOCs/year [16] to a maximum of 18.2 VOCs/year [17]. Overall, this SLR identified 95 studies, 39 of which reported frequency of VOCs and 13 of which reported frequency of complicated VOCs. Almost half of studies (43 out of 95) that reported VOC outcomes did not report a time period for the VOC outcomes. These studies are presented in the Appendix but were not included for data analysis as the number of VOCs is not interpretable without a time period. Among the 52 studies included for data analysis, 14 were conference abstracts which had sparse information on study methodology, including VOC definition. Congress abstracts were included in this SLR to supplement the limited peer-reviewed literature base. Studies included in the analysis were a mix of retrospective observational, prospective observational, and cross-sectional study designs.

The majority of studies identified in this SLR only considered VOCs managed in an inpatient or outpatient setting. Other evidence suggest that the frequency of VOCs is likely to be undercounted, as a large proportion (51–79%) of individuals with SCD report treating VOCs at home [11, 12]. Additionally, many patients report experiencing at least one barrier to receiving SCD-related healthcare services, citing barriers such as discrimination by health professionals, limited SCD centers, and difficulty trusting healthcare professionals, which can also contribute to VOCs being undercounted [12]. Among those studies in this SLR that included self-reported VOCs or those managed in a home setting, the mean annualized frequency was generally higher, ranging from 5.2 to 13.6 VOCs/year [19–21]. This is also consistent with a recent prospective cohort study published after the search period for this SLR, in which 35 U.S. patients were followed for 6 months and reported a median annualized frequency of 8 VOCs/year, with a mean duration of 2.7 days and 62.3% of VOC events self-managed at home [50]. Patients self-reported VOCs in their daily pain diary that were managed at home, in a clinic, in the emergency department, or in the hospital. This higher annualized frequency, 8 VOCs/year, is consistent with the range of frequencies, 5.2–13.6 VOCs/year, reported in other studies we identified in the SLR which considered self-reported VOCs or those self-managed in a home setting [19–21].

Few studies reported measures of VOC severity including the frequency of complicated VOCs, such as ACS, priapism, and hepatic/splenic sequestration. Studies that did report complicated VOCs found a frequency of < 1 per year. Only 1 study reported the duration of VOC episodes which ranged from 1.9 days (outpatient) to 11.7 days (inpatient) depending on treatment setting [39]. Given that many VOCs are managed at home, additional evidence is needed to assess the severity of VOCs. For example, it remains uncertain whether VOCs managed at home are actually less severe than those treated in an outpatient or inpatient setting, or merely under-reported. Furthermore, there is no well-accepted definition of overall SCD severity and VOC severity [51]. For this reason, some have advocated a shift away from the term VOC in favor of “acute pain episode", which would likely reveal the incidence to be even greater, not only by eliminating the criterion for healthcare utilization, but also reducing the dependence on subjective scales of severity. Results from this SLR support the need for application of a consistent definition of VOCs in clinical research, as well as for consistent measurement and reporting of the complicated VOCs [52].

### Limitations

The findings in the current SLR are subject to several limitations. Literature reviews are inherently limited by the accuracy of search terms used, which, in part, reflects the consistency in nomenclature and language used to describe the condition. While every attempt was made to use broad and effective search terms, and database search was supplemented by manual searches, some relevant studies can still be missed. In the context of a review of studies reporting VOC frequency, aggregating or comparing results is limited by variation in study designs and VOC definition/measurement standards, such that results are not necessarily directly comparable given these methodological differences. Although ranges were provided, statistical analysis or comparisons may be misleading given this heterogeneity. The study was not designed to assess the effect on interventions on VOCs. The SLR did not analyze concomitant treatments such as hydroxyurea and the differences in VOC frequency based on concomitant treatments. Due to the broad range of study designs included in the SLR (cross-sectional surveys, prospective studies, retrospective studies), treatment information reported among the 52 studies was variable and often limited, with more than a third of studies not reporting any form of treatment information (18/52; 34.6%). In addition, when treatment information for SCD was reported, studies varied in when the treatment was received (e.g., at baseline, during the study period, during VOCs only) and the types of treatments received (e.g., hydroxyurea only, multiple treatments including hydroxyurea, transfusions). International variability in treatment availability and clinical guidelines also likely affected treatment choice among patients with SCD. Finally, this SLR excluded clinical trials in order to study the frequency of VOCs in the real world independent of any particular treatment. Future real-world evidence is likely to include recently approved interventions known to reduce VOC frequency, potentially enabling research to assess comparative effectiveness of various treatments that reduce the rate of VOCs in real-world populations.

## Conclusions

To the best of our knowledge, this study is the first to systematically assess and synthesize literature on the frequency of VOCs in SCD in real-world settings. The SLR identified a wide range of definition of VOC and frequency of VOC experienced in real-world settings, with a majority of studies considering VOCs managed in an inpatient or outpatient setting. However, VOCs are often self-managed in the home care setting, and, among studies that did assess VOCs managed at home, frequency of VOCs was found to be higher. Measures of VOC severity were limited, with few studies that measured complicated VOCs and only 1 study reporting the duration of a VOC episode. This SLR highlights the need for consistent reporting of (1) self-reported VOCs, including those managed at home, (2) definitions of VOCs, (3) complicated VOCs, and (4) duration of VOC episodes in literature. Such research will increase the understanding of the impact of VOCs on individuals with SCD and enable caregivers to more effectively manage this serious disease.

## Supplementary Information

**Additional file 1.** Complete search strategies. This file contains detailed search strategies for MEDLINE database and listing of included congress abstract databases.

**Additional file 2.** Inclusion and exclusion criteria. This table presents inclusion and exclusion criteria for both database and additional supplementary manual searches of congresses.

**Additional file 3.** Summary of publications which reported frequency or prevalence of VOC without timing. This table presents publication information for all studies that did not report timing of VOCs or VOC-related outcomes.

**Additional file 4.** Summary of included publications. This table presents publication information and VOC definition as reported by the original study authors.

**Additional file 5.** Summary of reported treatment information in publications. This table presents reported treatment information for all studies.

## Data Availability

Detailed search strategy, inclusion and exclusion criteria, and list of included studies can be found in the supplementary information available with the online version of this article. For all other original data, please contact taiji.wang@analysisgroup.com.

## Abbreviations

ACS: Acute chest syndrome
ED: Emergency department
HRQoL: Health-related quality of life
IP: Inpatient
ISPOR: International society of pharmacoeconomics and outcomes research
OP: Outpatient
QoL: Quality of life
SCD: Sickle cell disease
SLR: Systematic literature review
SWAY: Sickle cell world assessment survey
VOC: Vaso-occlusive crisis
