# Supplementary material for: A systematic literature review of frequency of vaso-occlusive crises in sickle cell disease
Source: Orphanet J Rare Dis. 2021 Nov 2;16:460. doi: 10.1186/s13023-021-02096-6 (PMC8561926; doi:10.1186/s13023-021-02096-6)
Supplement: Supplementary file 1 — Additional file 1. Complete search strategies. This file contains detailed search strategies for MEDLINE database and listing of included congress abstract databases. [file 13023_2021_2096_MOESM1_ESM.docx]

# Additional file 1. Complete search strategies

Searches conducted on: June 15, 2020–June 30, 2020.

MEDLINE and MEDLINE In-Process Search Strategy (via OvidSp)

| # | Search terms | # hits |
| --- | --- | --- |

| *Disease terms* | |  |
| --- | --- | --- |
| 1 | exp Anemia/ | 160,552 |
| 2 | (sickle cell adj (anemia or anaemia or disease or crisis or hemoglobinopath)).ti,ab. | 20,395 |
| 3 | 1 or 2 | 165,226 |
| *VOC terms* | |  |
| 4 | (pain cris#s or vaso?occlusive cris#s or VOC or SCPC).af | 6551 |
| 5 | 3 and 4 | 885 |
| 6 | exp Priapism/ | 2212 |
| 7 | (erectile dysfunction or sexual dysfunction or penile dysfunction).ti,ab. | 24,079 |
| 8 | 6 or 7 | 26,056 |
| 9 | 3 and 8 | 411 |
| 10 | (acute chest syndrome or ACS).ti,ab. | 22,314 |
| 11 | 3 and 10 | 1045 |
| 12 | (splenic sequestration or hepatic sequestration).ti,ab. | 546 |
| 13 | 3 and 12 | 320 |
| 14 | 5 or 9 or 11 or 13 | 2426 |
| *General terms* | |  |
| 15 | Animals/ not Humans/ | 4,673,700 |
| 16 | 14 not 15 | 2387 |
| 17 | limit 16 to English language | 2245 |
| 18 | limit 17 to (case report or comment or editorial or letter) | 480 |
| 19 | 17 not 18 | 1765 |
| 20 | limit 19 to yr=”2000-Current” | 1408 |

Included Congresses (via Manual Search)

- **American Society of Hematology (ASH)**
- **American Society of Pediatric Hematology/Oncology (ASPHO)**
- **Academy of Managed Care Pharmacy (AMCP)**
- **European Hematology Association (EHA)**
- **Foundation for Sickle Cell Disease Research (FSCDR)**
- **International Society for Pharmacoeconomics and Outcomes Research (ISPOR)**
