# Supplementary material for: A systematic literature review of frequency of vaso-occlusive crises in sickle cell disease
Source: Orphanet J Rare Dis. 2021 Nov 2;16:460. doi: 10.1186/s13023-021-02096-6 (PMC8561926; doi:10.1186/s13023-021-02096-6)
Supplement: Supplementary file 2 — Additional file 2. Inclusion and exclusion criteria. This table presents inclusion and exclusion criteria for both database and additional supplementary manual searches of congresses. [file 13023_2021_2096_MOESM2_ESM.docx]

# Additional file 2. Inclusion and exclusion criteria

|  | Inclusion criteria | Exclusion criteria |
| --- | --- | --- |
| Population | Adult and pediatric patients with SCD | - Studies with mixed populations without outcomes reported separately for a SCD subgroup - Animal studies |
| Interventions | Any intervention, pharmacological or non-pharmacological, used in the treatment or management of SCD or none | N/A |
| Comparators | Any or none | N/A |
| Outcomes | - Frequency/prevalence of VOCs/pain crises - Frequency/prevalence of complicated VOCs (i.e., priapism, acute chest syndrome, splenic and hepatic sequestration) | No VOCs or VOC-related outcomes |
| Study Type | - Real-world studies   - Observational   - Prospective and retrospective cohorts   - Cross-sectional studies   - Data registries   - Administrative data, medical charts, or electronic health records   - Surveys - SLRs | - Clinical trials and retrospective analyses of clinical trials - SCD patient N < 15 - Narrative review articles - Case reports, editorials, letters - Recommendations/guidelines - Methods articles/protocols - Non-human studies - Earlier publications of an already included study by same authors with identical SCD populations and outcomes |
| Other | - English only - Any study geography - Published on or after 2000/01/01 | - Non-English - No abstract available - Published before 2000/01/01 |
