# Supplementary material for: A systematic literature review of frequency of vaso-occlusive crises in sickle cell disease
Source: Orphanet J Rare Dis. 2021 Nov 2;16:460. doi: 10.1186/s13023-021-02096-6 (PMC8561926; doi:10.1186/s13023-021-02096-6)
Supplement: Supplementary file 3 — Additional file 3. Summary of publications which reported frequency or prevalence of VOC without timing. This table presents publication information for all studies that did not report timing of VOCs or VOC-related outcomes. [file 13023_2021_2096_MOESM3_ESM.docx]

# Additional file 3. Summary of publications which reported frequency or prevalence of VOC without timing

| Study Identifier | Title | Journal Name/ Conference | Publication Year | Reference |
| --- | --- | --- | --- | --- |
| Adekile, 2017^1^ | The sickle beta-thalassemia phenotype | Journal of Pediatric Hematology/Oncology | 2017 | 39(5): 327-331 |
| Adeyoju, 2002^2^ | Priapism in sickle-cell disease: incidence, risk factors and complications - An international multicenter study | BJU International | 2002 | 90(9): 898-902 |
| Al-Ghazaly, 2013^3^ | Characteristics of sickle cell anemia in Yemen | Hemoglobin | 2013 | 37(1): 1-15 |
| Al-Hawsawi, 2004^4^ | Acute chest syndrome in sickle cell disease | Saudi Medical Journal | 2004 | 25(1): 116-117 |
| Alkindi, 2018^5^ | Predictors of adverse outcome in sickle cell disease patients from Oman | ASH 2018 | 2018 | — |
| Allali, 2019^6^ | Hepatobiliary complications in children with sickle cell disease: a retrospective review of medical records from 616 patients | Journal of Clinical Medicine | 2019 | 8(9): 18 |
| Al-Rimawi, 2006^7^ | Acute splenic sequestration in female children with sickle cell disease in the North of Jordan | Journal of Tropical Pediatrics | 2006 | 52(6): 416-420 |
| Alvaia, 2020^8^ | Prevalence of priapism in individuals with sickle cell disease and implications on male sexual function | Einstein | 2020 | 18: eAO5070 |
| Arduini, 2018^9^ | Prevalence and characteristics of priapism in sickle cell disease | Hemoglobin | 2018 | 42(2): 73-77 |
| Bardakdjian-Michau, 2002^10^ | Decreased morbidity in homozygous sickle cell disease detected at birth | Hemoglobin | 2002 | 26(3): 211-217 |
| Belisario, 2020^11^ | Hb Sbeta-Thalassemia in the REDS-III Brazil Sickle Cell Disease Cohort: clinical, laboratory and molecular characteristics | Hemoglobin | 2020 | 44(1): 1-9 |
| Ben Khaled, 2020^12^ | Prevalence and predictive factors of splenic sequestration crisis among 423 pediatric patients with sickle cell disease in Tunisia | Blood Cells Molecules & Diseases | 2020 | 80: 102374 |
| Benites, 2016^13^ | Sickle cell/beta-thalassemia: comparison of Sbeta^0^ and Sbeta^+^ Brazilian patients followed at a single institution | Hemoglobin | 2016 | 21(10): 623-629 |
| Brown, 2019^14^ | Beyond pain: the symptoms and impacts of sickle cell disease on children and their caregivers | FSCDR 2019 | 2019 | — |
| da Silva Filho, 2012^15^ | Sickle cell disease: acute clinical manifestations in early childhood and molecular characteristics in a group of children in Rio de Janeiro | Revista Brasileira de Hematologia e Hemoterapia | 2012 | 34(3): 196-201 |
| Dupervil, 2016^16^ | Emergency department visits and inpatient admissions associated with priapism among males with sickle cell disease in the United States, 2006-2010 | PLoS ONE [Electronic Resource] | 2016 | 11(4): e0153257 |
| Elliott, 2007^17^ | Genetic polymorphisms associated with priapism in sickle cell disease | British Journal of Haematology | 2007 | 137(3): 262-267 |
| El-Samahi, 2019^18^ | Sickle cell disease patients have normal neutrophil oxidative burst response | EHA 2019 | 2019 | — |
| Fisak, 2012^19^ | The relation between health-related quality of life, treatment adherence and disease severity in a paediatric sickle cell disease sample | Child: Care, Health & Development | 2012 | 38(2): 204-210 |
| Furtado, 2012^20^ | The prevalence of priapism in children and adolescents with sickle cell disease in Brazil | International Journal of Hematology | 2012 | 95(6): 648-651 |
| Gbadoe, 2001^21^ | Priapism in sickle cell anemia in Togo: prevalence and knowledge of this complication | Hemoglobin | 2001 | 25(4): 355-361 |
| Herquelot, 2019^22^ | Marginal structural model for studying the casual effect between vaso-occlusive crises and occurrence of death or complications in the sickle-cell disease patients | ISPOR EU 2019 | 2019 | — |
| Idris, 2019^23^ | Cross sectional survey of priapism and sexual dysfunction in 353 men with sickle cell disease | ASH 2019 | 2019 | — |
| Isa, 2020^24^ | Sickle cell disease clinical phenotypes in Nigeria: a preliminary analysis of the Sickle Pan Africa Research Consortium Nigeria database | Blood Cells Molecules & Diseases | 2020 | 84: 102438 |
| Jones, 2019^25^ | A clinical pathway reduces admissions for vaso-occlusive pain in sickle cell disease | ASH 2019 | 2019 | — |
| Kayle, 2019^26^ | Transition to adult care in sickle cell disease: a longitudinal study of clinical characteristics and disease severity | Pediatric Blood & Cancer | 2019 | 66(1): e27463 |
| King, 2007^27^ | Newborn sickle cell disease screening: the Jamaican experience (1995-2006) | Journal of Medical Screening | 2007 | 14(3): 117-122 |
| Koduri, 2001^28^ | Hemoglobin S-C disease revisited: clinical study of 106 adults | American Journal of Hematology | 2001 | 68(4): 298-300 |
| Morrison, 2015^29^ | Is testosterone deficiency a possible risk factor for priapism associated with sickle-cell disease? | International Urology & Nephrology | 2015 | 47(1): 47-52 |
| Munaretto, 2019^30^ | Acute chest syndrome in children with sickle cell disease in Italy: results of a national survey from the Italian association of pediatric hematology oncology (AIEOP) | ASH 2019 | 2019 | — |
| Nellesen, 2019^31^ | A systematic literature review of the burden of ischemic priapism in patients with sickle cell disease | ASH 2019 | 2019 | — |
| Neonato, 2000^32^ | Acute clinical events in 299 homozygous sickle cell patients living in France. French Study Group on sickle cell disease | European Journal of Haematology | 2000 | 65(3): 155-164 |
| Nolan, 2018^33^ | Hemolytic, vaso-occlusive and renal complications of SCD: report from the central Missouri cohort | ASH 2018 | 2018 | — |
| Notarangelo, 2020^34^ | HbS/beta+ thalassemia: really a mild disease? A national survey from the AIEOP sickle cell disease study group with genotype‑phenotype correlation | European Journal of Haematology | 2020 | 104(3): 214-222 |
| Pahl, 2016^35^ | Acute chest syndrome in sickle cell disease: effect of genotype and asthma | Experimental Biology & Medicine | 2016 | 241(7): 745-758 |
| Rizio, 2019^36^ | Impact of vaso-occlusive crises on quality of life, healthcare resource utilization and work productivity in sickle cell disease patients | FSCDR 2019 | 2019 | — |
| Renoux, 2017^37^ | Alpha-thalassaemia promotes frequent vaso-occlusive crises in children with sickle cell anaemia through haemorheological changes | Pediatric Blood & Cancer | 2017 | 64(8) |
| Saidi, 2016^38^ | Complications of sickle cell anaemia in children in Northwestern Tanzania | Hematology | 2016 | 21(4): 248-256 |
| Subbannan, 2009^39^ | Acute splenic complications and implications of splenectomy in hemoglobin SC disease | European Journal of Haematology | 2009 | 83(3): 258-260 |
| Tarer, 2006^40^ | Sickle cell anemia in Guadeloupean children: pattern and prevalence of acute clinical events | European Journal of Haematology | 2006 | 76(3): 193-199 |
| Valavi, 2010^41^ | How to reach rapid diagnosis in sickle cell disease? | Iranian Journal of Pediatrics | 2010 | 20(1): 69-74 |
| Zanette, 2011^42^ | Sickle cell anemia: delayed diagnosis in Bahia, Brazil--a largely Afro-descendant population | Ethnicity & Disease | 2011 | 21(2): 243-247 |
| Zawar, 2005^43^ | Non-invasive detection of endothelial dysfunction in sickle cell disease by Doppler ultrasonography | Journal of the Association of Physicians of India | 2005 | 53: 677-680 |

Abbreviations: ASH, American Society of Hematology; EHA, European Hematology Association; EU, Europe; FSCDR, Foundation for Sickle Cell Disease Research; ISPOR, International Society for Pharmacoeconomics and Outcomes Research; US, United States.

# References

1. Adekile AD, Akbulut N, Azab AF, Al-Sharida S, Thomas D. The sickle beta-thalassemia phenotype. *J Pediatr Hematol Oncol*. 2017;39(5):327-331.

2. Adeyoju AB, Olujohungbe AB, Morris J, et al. Priapism in sickle-cell disease; incidence, risk factors and complications - an international multicentre study. *BJU Int*. 2002;90(9):898-902.

3. Al-Ghazaly J, Al-Dubai W, Abdullah M, Al-Mahagri A, Al-Gharasi L. Characteristics of sickle cell anemia in Yemen. *Hemoglobin*. 2013;37(1):1-15.

4. Al-Hawsawi ZM. Acute chest syndrome in sickle cell disease. *Saudi Med J*. 2004;25(1):116-117.

5. Alkindi S, Al Jadidi S, Al Adawi S, Pathare A. Predictors of adverse outcome in sickle cell disease patients from Oman [abstract]. Presented at: 60th American Society of Hematology Annual Meeting and Exposition; San Diego, CA, USA; December 1-4, 2018.; 2018.

6. Allali S, de Montalembert M, Brousse V, et al. Hepatobiliary complications in children with sickle cell disease: a retrospective review of medical records from 616 patients. *Journal of Clinical Medicine*. 2019;8(9):18.

7. Al-Rimawi HS, Abdul-Qader M, Jallad MF, Amarin ZO. Acute splenic sequestration in female children with sickle cell disease in the North of Jordan. *J Trop Pediatr*. 2006;52(6):416-420.

8. Alvaia MA, Maia H, Nelli AM, et al. Prevalence of priapism in individuals with sickle cell disease and implications on male sexual function. *Einstein*. 2020;18:eAO5070.

9. Arduini GAO, Trovo de Marqui AB. Prevalence and characteristics of priapism in sickle cell disease. *Hemoglobin*. 2018;42(2):73-77.

10. Bardakdjian-Michau J, Guilloud-Batailie M, Maier-Redelsperger M, et al. Decreased morbidity in homozygous sickle cell disease detected at birth. *Hemoglobin*. 2002;26(3):211-217.

11. Belisario AR, Carneiro-Proietti AB, Sabino EC, et al. Hb Sbeta-Thalassemia in the REDS-III Brazil Sickle Cell Disease Cohort: clinical, laboratory and molecular characteristics. *Hemoglobin*. 2020;44(1):1-9.

12. Ben Khaled M, Ouederni M, Mankai Y, et al. Prevalence and predictive factors of splenic sequestration crisis among 423 pediatric patients with sickle cell disease in Tunisia. *Blood Cells Molecules & Diseases*. 2020;80:102374.

13. Benites BD, Bastos SO, Baldanzi G, et al. Sickle cell/beta-thalassemia: comparison of Sbeta0 and Sbeta+ Brazilian patients followed at a single institution. *Hematology*. 2016;21(10):623-629.

14. Brown C, Callaghan M, Clifford S, et al. Beyond pain: the symptoms and impacts of sickle cell disease on children and their caregivers [abstract]. Presented at: 13th Annual Sickle Cell Disease Research & Educational Symposium; Washington, DC, USA; June 7-9, 2019.; 2019.

15. da Silva Filho IL, Ribeiro GS, Moura PG, Vechi ML, Cavalcante AC, de Andrada-Serpa MJ. Sickle cell disease: acute clinical manifestations in early childhood and molecular characteristics in a group of children in Rio de Janeiro. *Revista Brasileira de Hematologia e Hemoterapia*. 2012;34(3):196-201.

16. Dupervil B, Grosse S, Burnett A, Parker C. Emergency department visits and inpatient admissions associated with priapism among males with sickle cell disease in the United States, 2006-2010. *PLoS ONE [Electronic Resource]*. 2016;11(4):e0153257.

17. Elliott L, Ashley-Koch AE, De Castro L, et al. Genetic polymorphisms associated with priapism in sickle cell disease. *Br J Haematol*. 2007;137(3):262-267.

18. El-Samahi M, Makkeyah S, Hamza M, Abouelnour A. Sickle cell disease patients have normal neutrophil oxidative burst response [abstract]. Presented at: 24th Congress of the European Hematology Association; Amsterdam, The Netherlands; June 13-16, 2019.; 2019.

19. Fisak B, Belkin MH, von Lehe AC, Bansal MM. The relation between health-related quality of life, treatment adherence and disease severity in a paediatric sickle cell disease sample. *Child Care Health Dev*. 2012;38(2):204-210.

20. Furtado PS, Costa MP, Ribeiro do Prado Valladares F, et al. The prevalence of priapism in children and adolescents with sickle cell disease in Brazil. *Int J Hematol*. 2012;95(6):648-651.

21. Gbadoe ADD, A. Segbena, A. Y. Nyadanu, M. Atakouma, Y. Kusiaku, K. Vovor, A. Assimadi, J. K. Priapism in sickle cell anemia in Togo: prevalence and knowledge of this complication. *Hemoglobin*. 2001;25(4):355-361.

22. Herquelot E, Morgan G, Lamarsalle L, et al. Marginal structural model for studying the casual effect between vaso-occlusive crises and occurrence of death or complications in the sickle-cell disease patients [abstract]. Presented at: International Society for Pharmaeconomics and Outcomes Research Europe; Copenhagen, Denmark; November 2-6, 2019. ; 2019.

23. Idris IM, Galadanci JA, Abba A, et al. Cross sectional survey of priapism and sexual dysfunction in 353 men with sickle cell disease [abstract]. Presented at: 61st American Society of Hematology Annual Meeting and Exposition; Orlando, FL, USA; December 7-10, 2019.; 2019.

24. Isa H, Adegoke S, Madu A, et al. Sickle cell disease clinical phenotypes in Nigeria: A preliminary analysis of the Sickle Pan Africa Research Consortium Nigeria database. *Blood Cells Molecules & Diseases*. 2020;84:102438.

25. Jones W, Myers L, Dasgupta A, Debord J. A clinical pathway reduces admissions for vaso-occlusive pain in sickle cell disease [abstract]. Presented at: 61st American Society of Hematology Annual Meeting and Exposition; Orlando, FL, USA; December 7-10, 2019.; 2019.

26. Kayle M, Docherty SL, Sloane R, et al. Transition to adult care in sickle cell disease: a longitudinal study of clinical characteristics and disease severity. *Pediatr Blood Cancer*. 2019;66(1):e27463.

27. King L, Fraser R, Forbes M, Grindley M, Ali S, Reid M. Newborn sickle cell disease screening: the Jamaican experience (1995-2006). *J Med Screen*. 2007;14(3):117-122.

28. Koduri PR, Agbemadzo B, Nathan S. Hemoglobin S-C disease revisited: clinical study of 106 adults. *Am J Hematol*. 2001;68(4):298-300.

29. Morrison BF, Anele UA, Reid ME, Madden WA, Feng Z, Burnett AL. Is testosterone deficiency a possible risk factor for priapism associated with sickle-cell disease? *Int Urol Nephrol*. 2015;47(1):47-52.

30. Munaretto V, Colombatti R, Tripodi SI, et al. Acute chest syndrome in children with sickle cell disease in Italy: results of a national survey from the Italian association of pediatric hematology oncology (AIEOP) [abstract]. Presented at: 61st American Society of Hematology Annual Meeting and Exposition; Orlando, FL, USA; December 7-10, 2019.; 2019.

31. Nellesen D, Lucas S, Liu CR, Bhor M, Paulose J, Burnett A. A systematic literature review of the burden of ischemic priapism in patients with sickle cell disease [abstract]. Presented at: 61st American Society of Hematology Annual Meeting and Exposition; Orlando, FL, USA; December 7-10, 2019.; 2019.

32. Neonato MG, Guilloud-Bataille M, Beauvais P, et al. Acute clinical events in 299 homozygous sickle cell patients living in France. French Study Group on Sickle Cell Disease. *Eur J Haematol*. 2000;65(3):155-164.

33. Nolan LW, Yoshida Y, Coberly E, Sathi BK. Hemolytic, vaso-occlusive and renal complications of SCD: report from the central missouri cohort [abstract]. Presented at: 60st American Society of Hematology Annual Meeting and Exposition; San Diego, CA, USA; December 1-4, 2018.; 2018.

34. Notarangelo LD, Agostini A, Casale M, et al. HbS/beta+ thalassemia: really a mild disease? A national survey from the AIEOP sickle Cell Disease Study Group with genotype-phenotype correlation. *Eur J Haematol*. 2020;104(3):214-222.

35. Pahl K, Mullen CA. Original Research: acute chest syndrome in sickle cell disease: effect of genotype and asthma. *Exp Biol Med*. 2016;241(7):745-758.

36. Rizio A, Bhor M, Lin X, et al. Impact of vaso-occlusive crises on quality of life, healthcare resource utilization and work productivity in sickle cell disease patients [abstract]. Presented at: 13th Annual Sickle Cell Disease Research & Educational Symposium; Washington, DC, USA; June 7-9, 2019.; 2019.

37. Renoux C, Connes P, Nader E, et al. Alpha-thalassaemia promotes frequent vaso-occlusive crises in children with sickle cell anaemia through haemorheological changes. *Pediatr Blood Cancer*. 2017;64(8).

38. Saidi H, Smart LR, Kamugisha E, et al. Complications of sickle cell anaemia in children in Northwestern Tanzania. *Hematology*. 2016;21(4):248-256.

39. Subbannan K, Ustun C, Natarajan K, et al. Acute splenic complications and implications of splenectomy in hemoglobin SC disease. *Eur J Haematol*. 2009;83(3):258-260.

40. Tarer V, Etienne-Julan M, Diara JP, et al. Sickle cell anemia in Guadeloupean children: pattern and prevalence of acute clinical events. *Eur J Haematol*. 2006;76(3):193-199.

41. Valavi E, Ansari MJ, Zandian K. How to reach rapid diagnosis in sickle cell disease? *Iranian Journal of Pediatrics*. 2010;20(1):69-74.

42. Zanette AM, Goncalves Mde S, Bahia RC, Nogueira LV, Arruda SM. Sickle cell anemia: delayed diagnosis in Bahia, Brazil--a largely Afro-descendant population. *Ethn Dis*. 2011;21(2):243-247.

43. Zawar SD, Vyawahare MA, Nerkar M, Jawahirani AR. Non-invasive detection of endothelial dysfunction in sickle cell disease by Doppler ultrasonography. *J Assoc Physicians India*. 2005;53:677-680.
