# Supplementary material for: A systematic literature review of frequency of vaso-occlusive crises in sickle cell disease
Source: Orphanet J Rare Dis. 2021 Nov 2;16:460. doi: 10.1186/s13023-021-02096-6 (PMC8561926; doi:10.1186/s13023-021-02096-6)
Supplement: Supplementary file 4 — Additional file 4. Summary of included publications. This table presents publication information and VOC definition as reported by the original study authors. [file 13023_2021_2096_MOESM4_ESM.docx]

**Additional file 4.** **Summary of included publications**

| Study Identifier | Title | Journal Name/ Conference | Publication Year | Reference | VOC Definition  (As Reported by Original Study Authors)^a^ |
| --- | --- | --- | --- | --- | --- |
| Adekile, 2019[1] | The sub-phenotypes of sickle cell disease in Kuwait | Hemoglobin | 2019 | 43(2): 83-87 | NR |
| Aloni, 2014[2] | Challenge of managing sickle cell disease in a pediatric population living in Kinshasa, Democratic Republic of Congo: a sickle cell center experience | Hemoglobin | 2014 | 38(3): 196-200 | Pain in the extremities, back, abdomen, chest, or head for which there was no other explanation (osteomyelitis, appendicitis) and required hospitalization for more than 48 hours |
| Alsultan, 2012[3] | Sickle cell disease subphenotypes in patients from Southwestern Province of Saudi Arabia | Journal of Pediatric Hematology/ Oncology | 2012 | 34: 79–84 | Number of acute VOC events occurred in the previous year including events treated in the outpatient setting |
| Andemariam, 2020[4] | Management strategies and satisfaction levels in patients with sickle cell disease in the US: interim results from the international sickle cell world assessment survey (SWAY) | FSCDR | 2020 | — | NR |
| Asnani, 2017[5] | Socio-environmental exposures and health outcomes among persons with sickle cell disease | PLoS ONE [Electronic Resource] | 2017 | 12(4): e0175260 | NR |
| Bailey, 2019[6] | Relationship between vaso-occlusive crises and important complications in sickle cell disease patients | ASH 2019 | 2019 | 134: 2167 | NR |
| Barner, 2019[7] | Association between hydroxyurea adherence and persistence and vaso-occlusive crises among Texas Medicaid recipients with sickle cell disease | AMCP 2019 | 2019 | 25(3): S34 | NR |
| Boyd, 2006[8] | Asthma is associated with acute chest syndrome and pain in children with sickle cell anemia | Blood | 2006 | 108: 2923-2927 | Pain in the extremities, back, abdomen, chest, or head for which no explanation other than SCA could be found, lasting at least 2 hours, leading to a clinic visit, and which was not classified as one of the following: skeletal/joint events, ACS, right upper quadrant pain, dactylitis, neurologic events, anemic episodes, febrile illness, and priapism |
| Bronte-Hall, 2020[9] | Real-world clinical burden of sickle cell disease in the US community-practice setting: a single-center experience from the foundation for sickle cell disease research | FSCDR 2020 | 2020 | — | NR |
| Brousse, 2018[10] | Prognostic factors of disease severity in infants with sickle cell anemia: a comprehensive longitudinal cohort study | American Journal of Hematology | 2018 | 93(11): 1411-1419 | Pain in the extremities, back, abdomen, chest, or head for which no other explanation could be found |
| Ceglie, 2019[11] | Gender-related differences in sickle cell disease in a pediatric cohort: a single-center retrospective study | Frontiers in Molecular Biosciences | 2019 | 6: 140 | Painful crises treated in other hospitals and those not needing hospitalization were excluded |
| Conneely, 2020[12] | Splenic complications in sickle cell disease: a retrospective cohort review | ASPHO 2020 | 2020 | — | NR |
| Darbari, 2013[13] | Severe painful vaso-occlusive crises and mortality in a contemporary adult sickle cell anemia cohort study | PLoS ONE [Electronic Resource] | 2013 | 8(11): e79923 | Low VOC (0 emergency department visits/hospitalizations for pain) and high VOC (≥1 events during the prior 12 months) groups |
| Darbari, 2012[14] | Markers of severe vaso-occlusive painful episode frequency in children and adolescents with sickle cell anemia | Journal of Pediatrics | 2012 | 160(2): 286-290 | Episodes of pain that may or may not have required pain medicines but did not prevent normal daily activity were described as mild pain episodes, and painful episodes requiring pain medications and changes in daily activities, such as missing work or school, were described as of moderate severity. Severe vaso-occlusive pain crisis included the episodes that required a visit to emergency department or doctor’s office or extremely severe painful episodes which required hospitalizations |
| Dave, 2019[15] | Attrition from care and clinical outcomes in a cohort of sickle cell disease patients in a tribal area of Western India | Tropical Medicine & Infectious Disease | 2019 | 4(4): 125 | NR |
| DeBaun, 2014[16] | Factors predicting future ACS episodes in children with sickle cell anemia | American Journal of Hematology | 2014 | 89(11): E212-E217 | An episode directly associated with SCA, which required hospitalization, and was treated with opioids |
| Delicou, 2019[17] | Sickle-cell disease in Greece: patient reported outcomes related to clinical complications, treatment choices and attitudes, beliefs and trends affecting potential participation in clinical trials - A Greek national multicentric study | ASH 2019 | 2019 | 134: 4838 | NR |
| Desai, 2019[18] | Clinical outcomes and healthcare utilization in patients with sickle cell disease: a nationwide cohort study of Medicaid beneficiaries | ASH 2019 | 2019 | 134: 3459 | NR |
| Donaldson, 2001[19] | Foetal haemoglobin in homozygous sickle cell disease: a study of patients with low HBF levels | Clinical & Laboratory Haematology | 2001 | 23(5): 285-289 | An episode of bone pain sufficient to interfere with function and require narcotic analgesia; events within 14 days were arbitrarily classed as the same episode |
| El Hoss, 2019[20] | Insights into determinants of spleen injury in sickle cell anemia | Blood Advances | 2019 | 3(15): 2328-2336 | NR |
| Elmariah, 2014[21] | Factors associated with survival in a contemporary adult sickle cell disease cohort | American Journal of Hematology | 2014 | 89(5): 530-535 | NR |
| Garadah, 2019[22] | The effects of hydroxyurea therapy on the six-minute walk distance in patients with adult sickle cell anemia: an echocardiographic study | Journal of Blood Medicine | 2019 | 10: 443-452 | NR |
| Hamdy, 2018[23] | Vitamin D and nonskeletal complications among Egyptian sickle cell disease patients | Advances in Hematology | 2018 | 3867283 | Mild, moderate, and severe where mild and moderate VOC were managed at home (with non-steroidal anti-inflammatory drugs and weak opioid, respectively) while severe VOC required hospitalization and the use of strong opioid |
| Inati, 2019[24] | Sickle cell disease burden in North Lebanon | ASH 2019 | 2019 | 134: 1022 | VOC events defined as the composite of pain |
| Jain, 2016[25] | Clinical events in a large prospective cohort of children with sickle cell disease in Nagpur, India: evidence against a milder clinical phenotype in India | Pediatric Blood & Cancer | 2016 | 63: 1814-1821 | Pain in the extremities, back, abdomen, chest, or head for which no other explanation could be found |
| Jaiyesimi, 2007[26] | Acute chest syndrome in Omani children with sickle cell disease: epidemiology and clinical profile | Annals of Tropical Paediatrics | 2007 | 27(3): 193-199 | NR |
| James, 2019[27] | Management strategies and satisfaction levels in patients with sickle cell disease: interim results from the international sickle cell world assessment survey (SWAY) | ASH 2019 | 2019 | 134: 1017 | NR |
| Janecek, 2019[28] | Integration of neuropsychology services in a sickle cell clinic and subsequent healthcare use for pain crises | Clinical Neuropsychologist | 2019 | 33(7):1195-1211 | NR |
| Joseph, 2019[29] | Vaso-occlusive crises and costs of sickle cell disease from a commercial payer's perspective | ASH 2019 | 2019 | 134: 3464 | A complicated VOC was defined as a VOC with a diagnosis of priapism, splenic sequestration, acute hepatic sequestration, or ACS. VOCs not meeting this definition were classified as uncomplicated |
| Knight-Madden, 2013[30] | Mortality, asthma, smoking and acute chest syndrome in young adults with sickle cell disease | Lung | 2013 | 191(1): 95-100 | NR |
| Lamarre, 2012[31] | Hemorheological risk factors of acute chest syndrome and painful vaso-occlusive crisis in children with sickle cell disease | Haematologica | 2012 | 97(11): 1641-1647 | An acute event was considered a VOC if the painful episode lasted for more than 4 h, the patient felt that the pain was typical of that of vaso-occlusion, no other etiology of pain could be identified by the physicians, and the patient was admitted to the Accident and Emergency Pediatric Department to treat the pain with parenteral opioids |
| Latremouille-Viau, 2020[32] | Vaso-occlusive crises and costs of sickle cell disease in Medicaid and Medicare beneficiaries: the perspective of public payers | AMCP 2020 | 2020 | 26(4a): S21 | NR |
| Le, 2018[33] | Is there an increase in sickle cell related events among the adult Belgian population? | ASH 2018 | 2018 | 132: 4923 | NR |
| Leleu, 2020[34] | Epidemiology and disease burden of SCD in France: a descriptive study based on a French nationwide claims database | EHA 2020 | 2020 | — | NR |
| Lettre, 2008[35] | DNA polymorphisms at the BCL11A, HBS1L-MYB, and beta-globin loci associate with fetal hemoglobin levels and pain crises in sickle cell disease | Proceedings of the National Academy of Sciences of the United States of America | 2008 | 105(33): 11869-11874 | NR |
| Lionnet, 2012[36] | Hemoglobin sickle cell disease complications: a clinical study of 179 cases | Haematologica | 2012 | 97(8): 1136-1141 | NR |
| Madu, 2014[37] | Priapism in homozygous sickle cell patients: important clinical and laboratory associations | Medical Principles & Practice | 2014 | 23(3): 259-263 | NR |
| McClish, 2006[38] | Gender differences in pain and healthcare utilization for adult sickle cell patients: the PiSCES project | Journal of Women's Health | 2006 | 15(2): 146-154 | A pain day was defined as a day with pain >0. A sickle cell crisis episode was defined as consecutive days in which the patient marked “crisis” on the diary |
| Neto, 2011[39] | The association of infection and clinical severity in sickle cell anaemia patients | Transactions of the Royal Society of Tropical Medicine & Hygiene | 2011 | 105(3): 121-126 | NR |
| Nimgaonkar, 2014[40] | Comprehensive integrated care for patients with sickle cell disease in a remote aboriginal tribal population in Southern India | Pediatric Blood & Cancer | 2014 | 61(4): 702-705 | NR |
| Osunkwo, 2020[41] | Incidence and management of vaso-occlusive crises in patients with sickle cell disease: a country and age analysis of the international sickle cell world assessment survey (SWAY) | EHA 2020 | 2020 | — | Severe pain crises |
| Rezende, 2018[42] | Clinical and hematological profile in a newborn cohort with hemoglobin SC | Jornal de Pediatria | 2018 | 94(6): 666-672 | NR |
| Rizio, 2020[43] | The relationship between frequency and severity of vaso-occlusive crises and health-related quality of life and work productivity in adults with sickle cell disease | Quality of Life Research | 2020 | 29(6): 1533-1547 | NR |
| Schuchard, 2019[44] | Hydroxyurea use in young infants with sickle cell disease | Pediatric Blood & Cancer | 2019 | 66(7): e27650 | NR |
| Shah, 2019[45] | Treatment patterns and economic burden of sickle-cell disease patients prescribed hydroxyurea: a retrospective claims-based study | Health & Quality of Life Outcomes | 2019 | 17(1): 155 | NR |
| Shah, 2019[46] | Sickle cell disease complications: Prevalence and resource utilization | PLoS ONE [Electronic Resource] | 2019 | 14(7): e0214355 | A complicated VOC episode was defined as the presence of a diagnosis of other SCD complications during the VOC episode, and uncomplicated VOC was defined as no occurrence of any other SCD complication during the VOC episode |
| Shome, 2016[47] | The effect of hydroxyurea therapy in Bahraini sickle cell disease patients | Indian Journal of Hematology & Blood Transfusion | 2016 | 32(1): 104-109 | Acute pain crisis episode |
| Upadhye, 2016[48] | Neonatal screening and the clinical outcome in children with sickle cell disease in Central India | PLoS ONE [Electronic Resource] | 2016 | 11(1): e0147081 | NR |
| van Tuijn, 2017[49] | Prospective evaluation of chronic organ damage in adult sickle cell patients: a seven-year follow-up study | American Journal of Hematology | 2017 | 92(10): E584-E590 | Occurrence of pain in the extremities, back, abdomen, chest, or head that led to a clinic visit and could not be explained otherwise |
| Willen, 2018[50] | Age is a predictor of a small decrease in lung function in children with sickle cell anemia | American Journal of Hematology | 2018 | 93(3): 408-415 | Hospitalization for SCA-associated pain, excluding headaches, and requiring opioid treatment |
| Willen, 2018[51] | Aeroallergen sensitization predicts acute chest syndrome in children with sickle cell anaemia | British Journal of Haematology | 2018 | 180(4): 571-577 | Hospitalization for SCA-associated pain, excluding headaches, and requiring opioid treatment |
| Yates, 2013[52] | Hydroxyurea treatment of children with hemoglobin SC disease | Pediatric Blood & Cancer | 2013 | 60(2): 323-325 | NR |

^a^ As reported by the original study authors. Reported definitions are as complete as possible and have not been edited.

Abbreviations: ACS, acute chest syndrome; AMCP, Academy of Managed Care Pharmacy; ASH, American Society of Hematology; ASPHO, American Society of Pediatric Hematology/Oncology; EHA, European Hematology Association; DNA, deoxyribonucleic acid; FSCDR, Foundation for Sickle Cell Disease Research; HBF, fetal hemoglobin; NR, not reported; SCD, sickle cell disease; US, United States; VOC, vaso-occlusive crisis.

**References**

1. Adekile AD, Al-Sherida S, Marouf R, Mustafa N, Thomas D. The sub-phenotypes of sickle cell disease in Kuwait. Hemoglobin. 2019;43(2):83-7. doi: <https://dx.doi.org/10.1080/03630269.2019.1610427>. PubMed PMID: 31144996.

2. Aloni MN, Nkee L. Challenge of managing sickle cell disease in a pediatric population living in Kinshasa, Democratic Republic of Congo: a sickle cell center experience. Hemoglobin. 2014;38(3):196-200. doi: <https://dx.doi.org/10.3109/03630269.2014.896810>. PubMed PMID: 24669956.

3. Alsultan A, Aleem A, Ghabbour H, AlGahtani FH, Al-Shehri A, Osman ME, et al. Sickle cell disease subphenotypes in patients from Southwestern Province of Saudi Arabia. J Pediatr Hematol Oncol. 2012;34(2):79-84. doi: <https://dx.doi.org/10.1097/MPH.0b013e3182422844>. PubMed PMID: 22322941.

4. Andemariam B, James J, Inusa B, El Rassi F, Francis-Gibson B, Nero A, et al., editors. Management strategies and satisfaction levels in patients with sickle cell disease in the US: interim results from the sickle cell world assessment survey (SWAY) [abstract]. Presented at: The Foundation for Sickle Cell Disease Research Congress; Virtual; June 2020.2020.

5. Asnani MR, Knight Madden J, Reid M, Greene LG, Lyew-Ayee P. Socio-environmental exposures and health outcomes among persons with sickle cell disease. PLoS ONE [Electronic Resource]. 2017;12(4):e0175260. doi: <https://dx.doi.org/10.1371/journal.pone.0175260>. PubMed PMID: 28384224.

6. Bailey M, Abioye A, Morgan G, Burke T, Disher T, Brown S, et al., editors. Relationship between vaso-occlusive crises and important complications in sickle cell disease patients [abstract]. Presented at: 61st American Society of Hematology Annual Meeting and Exposition; Orlando, FL, USA; December 7-10, 2019.2019.

7. Barner J, Kang H, Richards K, Bhor M, Paulose J, Kutlar A, editors. Association between hydroxyurea adherence and persistence and vaso-occlusive crises among Texas Medicaid recipients with sickle cell disease [abstract]. Academy of Managed Care Pharmacy Managed Care & Specialty Pharmacy Annual Meeting 2019; San Diego, CA, USA; March 25-28, 2019.2019.

8. Boyd JH, Macklin EA, Strunk RC, DeBaun MR. Asthma is associated with acute chest syndrome and pain in children with sickle cell anemia. Blood. 2006;108(9):2923-7. PubMed PMID: 16690969.

9. Bronte-Hall L, Parkin M, Green C, Tchouambou D, Huynh L, Puri-Sharma C, et al., editors. Real-world clinical burden of sickle cell disease in the US community-practice setting: a single-center experience from the foundation for sickle cell disease research [abstract]. Presented at: 14th Annual Sickle Cell Disease Research & Educational Symposium; Virtual; September 22-25, 2020.2020.

10. Brousse V, El Hoss S, Bouazza N, Arnaud C, Bernaudin F, Pellegrino B, et al. Prognostic factors of disease severity in infants with sickle cell anemia: a comprehensive longitudinal cohort study. Am J Hematol. 2018;93(11):1411-9. doi: <https://dx.doi.org/10.1002/ajh.25260>. PubMed PMID: 30132969.

11. Ceglie G, Di Mauro M, Tarissi De Jacobis I, de Gennaro F, Quaranta M, Baronci C, et al. Gender-related differences in sickle cell disease in a pediatric cohort: a single-center retrospective study. Front. 2019;6:140. doi: <https://dx.doi.org/10.3389/fmolb.2019.00140>. PubMed PMID: 31867340.

12. Conneely S, Mangum R, Lupo P, Scheurer M, George A, editors. Splenic complications in sickle cell disease: a retrospective cohort review [abstract]. Presented at: American Society of Pediatric Hematology/Oncology Conference; Virtual; May 2020.2020.

13. Darbari D, Wang Z, Kwak M, Hildesheim M, Nichols J, Allen D, et al. Severe painful vaso-occlusive crises and mortality in a contemporary adult sickle cell anemia cohort study. PLoS ONE [Electronic Resource]. 2013;8(11):e79923. doi: <https://dx.doi.org/10.1371/journal.pone.0079923>. PubMed PMID: 24224021.

14. Darbari D, Onyekwere O, Nouraie M, Minniti C, Luchtman-Jones L, Rana S, et al. Markers of severe vaso-occlusive painful episode frequency in children and adolescents with sickle cell anemia. J Pediatr. 2012;160(2):286-90. doi: <https://dx.doi.org/10.1016/j.jpeds.2011.07.018>. PubMed PMID: 21890147.

15. Dave K, Chinnakali P, Thekkur P, Desai S, Vora C, Desai G. Attrition from care and clinical outcomes in a cohort of sickle cell disease patients in a tribal area of Western India. Trop. 2019;4(4):01. doi: <https://dx.doi.org/10.3390/tropicalmed4040125>. PubMed PMID: 31581481.

16. DeBaun MR, Rodeghier M, Cohen R, Kirkham FJ, Rosen CL, Roberts I, et al. Factors predicting future ACS episodes in children with sickle cell anemia. Am J Hematol. 2014;89(11):E212-7. doi: <https://dx.doi.org/10.1002/ajh.23819>. PubMed PMID: 25088663.

17. Delicou S, Diamantidis M, Manganas K, Eftychiadis E, Pantelidou D, Kourakli A, et al., editors. Sickle-cell disease in Greece: patient reported outcomes related to clinical complications, treatment choices and attitudes, beliefs and trends affecting potential participation in clinical trials - A Greek national multicentric study [abstract]. Presented at: 61st American Society of Hematology Annual Meeting and Exposition; Orlando, FL, USA; December 7-10, 2019.2019.

18. Desai R, Mahesri M, Levin R, Globe D, McKerracher K, Mutebi A, et al., editors. Clinical outcomes and healthcare utilization in patients with sickle cell disease: a nationwide cohort study of Medicaid beneficiaries [abstract]. Presented at: 61st American Society of Hematology Annual Meeting and Exposition; Orlando, FL, USA; December 7-10, 2019.2019.

19. Donaldson A, Thomas P, Serjeant BE, Serjeant GR. Foetal haemoglobin in homozygous sickle cell disease: a study of patients with low HBF levels. Clin Lab Haematol. 2001;23(5):285-9. PubMed PMID: 11703409.

20. El Hoss S, Cochet S, Marin M, Lapoumeroulie C, Dussiot M, Bouazza N, et al. Insights into determinants of spleen injury in sickle cell anemia. Blood Adv. 2019;3(15):2328-36. doi: <https://dx.doi.org/10.1182/bloodadvances.2019000106>. PubMed PMID: 31391165.

21. Elmariah H, Garrett M, De Castro L, Jonassaint J, Ataga K, Eckman J, et al. Factors associated with survival in a contemporary adult sickle cell disease cohort. Am J Hematol. 2014;89(5):530-5. doi: <https://dx.doi.org/10.1002/ajh.23683>. PubMed PMID: 24478166.

22. Garadah T, Mandeel F, Jaradat A, Bin Thani K. The effects of hydroxyurea therapy on the six-minute walk distance in patients with adult sickle cell anemia: an echocardiographic study. J Blood Med. 2019;10:443-52. doi: <https://dx.doi.org/10.2147/JBM.S203828>. PubMed PMID: 31920416.

23. Hamdy M, Salama N, Maher G, Elrefaee A. Vitamin D and nonskeletal complications among Egyptian sickle cell disease patients. Adv Hematol. 2018;2018:3867283. doi: <https://dx.doi.org/10.1155/2018/3867283>. PubMed PMID: 30305813.

24. Inati A, Al Alam C, El Ojaimi C, Hamad T, Kanakamedala H, Pilipovic V, et al., editors. Sickle cell disease burden in North Lebanon [abstract]. Presented at: 61st American Society of Hematology Annual Meeting and Exposition; Orlando, FL, USA; December 7-10, 2019.2019.

25. Jain D, Arjunan A, Sarathi V, Jain H, Bhandarwar A, Vuga M, et al. Clinical events in a large prospective cohort of children with sickle cell disease in Nagpur, India: evidence against a milder clinical phenotype in India. Pediatr Blood Cancer. 2016;63(10):1814-21. doi: <https://dx.doi.org/10.1002/pbc.26085>. PubMed PMID: 27279568.

26. Jaiyesimi O, Kasem M. Acute chest syndrome in Omani children with sickle cell disease: epidemiology and clinical profile. Ann Trop Paediatr. 2007;27(3):193-9. PubMed PMID: 17716447.

27. James J, Andemariam B, Inusa B, El-Rassi F, Francis-Gibson B, Nero A, et al., editors. Management strategies and satisfaction levels in patients with sickle cell disease: interim results from the international sickle cell world assessment survey (SWAY) [abstract]. Presented at: 61st American Society of Hematology Annual Meeting and Exposition; Orlando, FL, USA; December 7-10, 2019.2019.

28. Janecek J, Dorociak K, Piper L, Kelleher T, Pliskin N, Gowhari M, et al. Integration of neuropsychology services in a sickle cell clinic and subsequent healthcare use for pain crises. Clin Neuropsychol. 2019;33(7):1195-211. doi: <https://dx.doi.org/10.1080/13854046.2018.1535664>. PubMed PMID: 30472925.

29. Joseph GJ, Latremouille-Viau D, Sharma VK, Gagnon-Sanschagrin P, Bhor M, Khare A, et al. Vaso-occlusive crises and costs of sickle cell disease from a commercial payer's perspective. Blood. 2019;134(Supplement_1):3464-. doi: 10.1182/blood-2019-124920.

30. Knight-Madden J, Barton-Gooden A, Weaver S, Reid M, Greenough A. Mortality, asthma, smoking and acute chest syndrome in young adults with sickle cell disease. Lung. 2013;191(1):95-100. doi: <https://dx.doi.org/10.1007/s00408-012-9435-3>. PubMed PMID: 23149803.

31. Lamarre Y, Romana M, Waltz X, Lalanne-Mistrih ML, Tressieres B, Divialle-Doumdo L, et al. Hemorheological risk factors of acute chest syndrome and painful vaso-occlusive crisis in children with sickle cell disease. Haematologica. 2012;97(11):1641-7. doi: <https://dx.doi.org/10.3324/haematol.2012.066670>. PubMed PMID: 22689686.

32. Latremouille-Viau D, Bhor M, Sharma V, Puckrein G, Gagnon-Sanschagrin P, Khare A, et al., editors. Vaso-occlusive crises and costs of sickle cell disease in Medicaid and Medicare beneficiaries: the perspective of public payers [abstract]. Academy of Managed Care Pharmacy Managed Care & Specialty Pharmacy Annual Meeting 2020; Virtual; April 2020.2020.

33. Le P, Gulbis B, Dedeken L, Rozen L, Vermylen C, Vanderfaeillie A, et al., editors. Is there an increase in sickle cell related events among the adult Belgian population [abstract]? Presented at: 60th American Society of Hematology Annual Meeting and Exposition; San Diego, CA USA; December 1-4, 2018.2018.

34. Leleu H, Arlet J, Habibi A, Etienne-Julan M, Pita M, Granghaud A, et al., editors. Epidemiology and disease burden of SCD in France: a descriptive study based on a French nationwide claims database [abstract]. Presented at: 26th Congress of the European Hematology Association; Virtual; June 20202020.

35. Lettre G, Sankaran V, Bezerra M, Araujo A, Uda M, Sanna S, et al. DNA polymorphisms at the BCL11A, HBS1L-MYB, and beta-globin loci associate with fetal hemoglobin levels and pain crises in sickle cell disease. Proc Natl Acad Sci U S A. 2008;105(33):11869-74. doi: <https://dx.doi.org/10.1073/pnas.0804799105>. PubMed PMID: 18667698.

36. Lionnet F, Hammoudi N, Stojanovic K, Avellino V, Grateau G, Girot R, et al. Hemoglobin sickle cell disease complications: a clinical study of 179 cases. Haematologica. 2012;97(8):1136-41. doi: <https://dx.doi.org/10.3324/haematol.2011.055202>. PubMed PMID: 22315500.

37. Madu AJ, Ubesie A, Ocheni S, Chinawa J, Madu KA, Ibegbulam OG, et al. Priapism in homozygous sickle cell patients: important clinical and laboratory associations. Med Princ Pract. 2014;23(3):259-63. doi: <https://dx.doi.org/10.1159/000360608>. PubMed PMID: 24685837.

38. McClish DK, Levenson JL, Penberthy LT, Roseff SD, Bovbjerg VE, Roberts JD, et al. Gender differences in pain and healthcare utilization for adult sickle cell patients: the PiSCES project. J Womens Health. 2006;15(2):146-54. PubMed PMID: 16536678.

39. Neto JP, Lyra IM, Reis MG, Goncalves MS. The association of infection and clinical severity in sickle cell anaemia patients. Trans R Soc Trop Med Hyg. 2011;105(3):121-6. doi: <https://dx.doi.org/10.1016/j.trstmh.2010.11.007>. PubMed PMID: 21216418.

40. Nimgaonkar V, Krishnamurti L, Prabhakar H, Menon N. Comprehensive integrated care for patients with sickle cell disease in a remote aboriginal tribal population in southern India. Pediatr Blood Cancer. 2014;61(4):702-5. doi: <https://dx.doi.org/10.1002/pbc.24723>. PubMed PMID: 24347362.

41. Osunkwo I, Andemariam B, Inusa B, El Rassi F, Francis-Gibson B, Nero A, et al., editors. Incidence and management of vaso-occlusive crises in patients with sickle cell disease: a country and age analysis of the international sickle cell world assessment survey (SWAY) [abstract]. Presented at: 26th Congress of the European Hematology Association; Virtual; June 20202020.

42. Rezende PVS, M. V. Campos, G. F. Vieira, L. L. M. Souza, M. B. Belisario, A. R. Silva, C. M. Viana, M. B. Clinical and hematological profile in a newborn cohort with hemoglobin SC. J Pediatr (Rio J). 2018;94(6):666-72. doi: <https://dx.doi.org/10.1016/j.jped.2017.09.010>. PubMed PMID: 29195085.

43. Rizio A, Bhor M, Lin X, McCausland KL, White MK, Paulose J, et al. The relationship between frequency and severity of vaso-occlusive crises and health-related quality of life and work productivity in adults with sickle cell disease. Qual Life Res. 2020;29(6):1533-47. doi: <https://dx.doi.org/10.1007/s11136-019-02412-5>. PubMed PMID: 31933113.

44. Schuchard SB, Lissick JR, Nickel A, Watson D, Moquist KL, Blaylark RM, et al. Hydroxyurea use in young infants with sickle cell disease. Pediatr Blood Cancer. 2019;66(7):e27650. doi: <https://dx.doi.org/10.1002/pbc.27650>. PubMed PMID: 30729675.

45. Shah N, Bhor M, Xie L, Halloway R, Arcona S, Paulose J, et al. Treatment patterns and economic burden of sickle-cell disease patients prescribed hydroxyurea: a retrospective claims-based study. Health Qual Life Outcomes. 2019;17(1):155. doi: <https://dx.doi.org/10.1186/s12955-019-1225-7>. PubMed PMID: 31619251.

46. Shah N, Bhor M, Xie L, Paulose J, Yuce H. Sickle cell disease complications: prevalence and resource utilization. PLoS ONE [Electronic Resource]. 2019;14(7):e0214355. doi: <https://dx.doi.org/10.1371/journal.pone.0214355>. PubMed PMID: 31276525.

47. Shome DK, Al Ajmi A, Radhi AA, Mansoor EJ, Majed KS. The effect of hydroxyurea therapy in Bahraini sickle cell disease patients. Indian J Hematol Blood Transfus. 2016;32(1):104-9. doi: <https://dx.doi.org/10.1007/s12288-015-0529-y>. PubMed PMID: 26855516.

48. Upadhye DS, Jain DL, Trivedi YL, Nadkarni AH, Ghosh K, Colah RB. Neonatal screening and the clinical outcome in children with sickle cell disease in Central India. PLoS ONE [Electronic Resource]. 2016;11(1):e0147081. doi: <https://dx.doi.org/10.1371/journal.pone.0147081>. PubMed PMID: 26785407.

49. van Tuijn CFJ, Schimmel M, van Beers EJ, Nur E, Biemond BJ. Prospective evaluation of chronic organ damage in adult sickle cell patients: a seven-year follow-up study. Am J Hematol. 2017;92(10):E584-E90. doi: <https://dx.doi.org/10.1002/ajh.24855>. PubMed PMID: 28699283.

50. Willen SM, Cohen R, Rodeghier M, Kirkham F, Redline SS, Rosen C, et al. Age is a predictor of a small decrease in lung function in children with sickle cell anemia. Am J Hematol. 2018;93(3):408-15. doi: <https://dx.doi.org/10.1002/ajh.25003>. PubMed PMID: 29226507.

51. Willen SM, Rodeghier M, Strunk RC, Bacharier LB, Rosen CL, Kirkham FJ, et al. Aeroallergen sensitization predicts acute chest syndrome in children with sickle cell anaemia. Br J Haematol. 2018;180(4):571-7. doi: <https://dx.doi.org/10.1111/bjh.15076>. PubMed PMID: 29363738.

52. Yates AM, Dedeken L, Smeltzer MP, Lebensburger JD, Wang WC, Robitaille N. Hydroxyurea treatment of children with hemoglobin SC disease. Pediatr Blood Cancer. 2013;60(2):323-5. doi: <https://dx.doi.org/10.1002/pbc.24283>. PubMed PMID: 22949140.
