# Supplementary material for: A systematic literature review of frequency of vaso-occlusive crises in sickle cell disease
Source: Orphanet J Rare Dis. 2021 Nov 2;16:460. doi: 10.1186/s13023-021-02096-6 (PMC8561926; doi:10.1186/s13023-021-02096-6)
Supplement: Supplementary file 5 — Additional file 5. Summary of reported treatment information in publications. This table presents reported treatment information for all studies. [file 13023_2021_2096_MOESM5_ESM.docx]

# Additional file 5. Summary of reported treatment information in publications

| **Treatment Information Reported** | **# of Publications** | **References** |
| --- | --- | --- |
| Not reported^a^ | 18 | [1-18] |
| Reported hydroxyurea use only^b^ | 12 | [19-30] |
| Reported multiple treatments (including hydroxyurea use)^c^ | 12 | [31-42] |
| Other treatment information reported^d^ | 10 | [43-52] |

^a^ Three studies did not report treatment information but did note that patients were excluded from enrollment if they were receiving hydroxyurea.[4, 7, 8]

^b^ All patients were receiving hydroxyurea for three studies.[24, 25, 28] One study compared patients receiving hydroxyurea to those not receiving hydroxyurea.[22] One study included patients with at least one prescription claim for hydroxyurea.[29]

^c^ Multiple treatments included folic acid, antibiotic prophylaxis, iron chelator, blood transfusions, opioid therapy, and other pain medications. One study reported multiple treatments but only included patients with at least one prescription claim for hydroxyurea.[37]

^d^ Other treatment information included reporting only blood transfusion information, pain crisis treatments, or other types of treatment programs. Additionally, two studies noted patients received “standard age-appropriate care”, and one study noted patients received no active management.[44, 47, 50]

**References**

1. Asnani MR, Knight Madden J, Reid M, Greene LG, Lyew-Ayee P. Socio-environmental exposures and health outcomes among persons with sickle cell disease. PLoS ONE [Electronic Resource]. 2017;12(4):e0175260. doi: <https://dx.doi.org/10.1371/journal.pone.0175260>. PubMed PMID: 28384224.

2. Donaldson A, Thomas P, Serjeant BE, Serjeant GR. Foetal haemoglobin in homozygous sickle cell disease: a study of patients with low HBF levels. Clin Lab Haematol. 2001;23(5):285-9. PubMed PMID: 11703409.

3. Hamdy M, Salama N, Maher G, Elrefaee A. Vitamin D and nonskeletal complications among Egyptian sickle cell disease patients. Adv Hematol. 2018;2018:3867283. doi: <https://dx.doi.org/10.1155/2018/3867283>. PubMed PMID: 30305813.

4. Jain D, Arjunan A, Sarathi V, Jain H, Bhandarwar A, Vuga M, et al. Clinical events in a large prospective cohort of children with sickle cell disease in Nagpur, India: evidence against a milder clinical phenotype in India. Pediatr Blood Cancer. 2016;63(10):1814-21. doi: <https://dx.doi.org/10.1002/pbc.26085>. PubMed PMID: 27279568.

5. Janecek J, Dorociak K, Piper L, Kelleher T, Pliskin N, Gowhari M, et al. Integration of neuropsychology services in a sickle cell clinic and subsequent healthcare use for pain crises. Clin Neuropsychol. 2019;33(7):1195-211. doi: <https://dx.doi.org/10.1080/13854046.2018.1535664>. PubMed PMID: 30472925.

6. Knight-Madden J, Barton-Gooden A, Weaver S, Reid M, Greenough A. Mortality, asthma, smoking and acute chest syndrome in young adults with sickle cell disease. Lung. 2013;191(1):95-100. doi: <https://dx.doi.org/10.1007/s00408-012-9435-3>. PubMed PMID: 23149803.

7. Lamarre Y, Romana M, Waltz X, Lalanne-Mistrih ML, Tressieres B, Divialle-Doumdo L, et al. Hemorheological risk factors of acute chest syndrome and painful vaso-occlusive crisis in children with sickle cell disease. Haematologica. 2012;97(11):1641-7. doi: <https://dx.doi.org/10.3324/haematol.2012.066670>. PubMed PMID: 22689686.

8. Lettre G, Sankaran V, Bezerra M, Araujo A, Uda M, Sanna S, et al. DNA polymorphisms at the BCL11A, HBS1L-MYB, and beta-globin loci associate with fetal hemoglobin levels and pain crises in sickle cell disease. Proc Natl Acad Sci U S A. 2008;105(33):11869-74. doi: <https://dx.doi.org/10.1073/pnas.0804799105>. PubMed PMID: 18667698.

9. Neto JP, Lyra IM, Reis MG, Goncalves MS. The association of infection and clinical severity in sickle cell anaemia patients. Trans R Soc Trop Med Hyg. 2011;105(3):121-6. doi: <https://dx.doi.org/10.1016/j.trstmh.2010.11.007>. PubMed PMID: 21216418.

10. Shah N, Bhor M, Xie L, Paulose J, Yuce H. Sickle cell disease complications: prevalence and resource utilization. PLoS ONE [Electronic Resource]. 2019;14(7):e0214355. doi: <https://dx.doi.org/10.1371/journal.pone.0214355>. PubMed PMID: 31276525.

11. Willen SM, Rodeghier M, Strunk RC, Bacharier LB, Rosen CL, Kirkham FJ, et al. Aeroallergen sensitization predicts acute chest syndrome in children with sickle cell anaemia. Br J Haematol. 2018;180(4):571-7. doi: <https://dx.doi.org/10.1111/bjh.15076>. PubMed PMID: 29363738.

12. Conneely S, Mangum R, Lupo P, Scheurer M, George A, editors. Splenic complications in sickle cell disease: a retrospective cohort review [abstract]. Presented at: American Society of Pediatric Hematology/Oncology Conference; Virtual; May 2020.2020.

13. Latremouille-Viau D, Bhor M, Sharma V, Puckrein G, Gagnon-Sanschagrin P, Khare A, et al., editors. Vaso-occlusive crises and costs of sickle cell disease in Medicaid and Medicare beneficiaries: the perspective of public payers [abstract]. Academy of Managed Care Pharmacy Managed Care & Specialty Pharmacy Annual Meeting 2020; Virtual; April 2020.2020.

14. Bailey M, Abioye A, Morgan G, Burke T, Disher T, Brown S, et al., editors. Relationship between vaso-occlusive crises and important complications in sickle cell disease patients [abstract]. Presented at: 61st American Society of Hematology Annual Meeting and Exposition; Orlando, FL, USA; December 7-10, 2019.2019.

15. Desai R, Mahesri M, Levin R, Globe D, McKerracher K, Mutebi A, et al., editors. Clinical outcomes and healthcare utilization in patients with sickle cell disease: a nationwide cohort study of Medicaid beneficiaries [abstract]. Presented at: 61st American Society of Hematology Annual Meeting and Exposition; Orlando, FL, USA; December 7-10, 2019.2019.

16. Osunkwo I, Andemariam B, Inusa B, El Rassi F, Francis-Gibson B, Nero A, et al., editors. Incidence and management of vaso-occlusive crises in patients with sickle cell disease: a country and age analysis of the international sickle cell world assessment survey (SWAY) [abstract]. Presented at: 26th Congress of the European Hematology Association; Virtual; June 20202020.

17. Joseph GJ, Latremouille-Viau D, Sharma VK, Gagnon-Sanschagrin P, Bhor M, Khare A, et al. Vaso-occlusive crises and costs of sickle cell disease from a commercial payer's perspective. Blood. 2019;134(Supplement_1):3464-. doi: 10.1182/blood-2019-124920.

18. Le P, Gulbis B, Dedeken L, Rozen L, Vermylen C, Vanderfaeillie A, et al., editors. Is there an increase in sickle cell related events among the adult Belgian population [abstract]? Presented at: 60th American Society of Hematology Annual Meeting and Exposition; San Diego, CA USA; December 1-4, 2018.2018.

19. Adekile AD, Al-Sherida S, Marouf R, Mustafa N, Thomas D. The sub-phenotypes of sickle cell disease in Kuwait. Hemoglobin. 2019;43(2):83-7. doi: <https://dx.doi.org/10.1080/03630269.2019.1610427>. PubMed PMID: 31144996.

20. Darbari D, Wang Z, Kwak M, Hildesheim M, Nichols J, Allen D, et al. Severe painful vaso-occlusive crises and mortality in a contemporary adult sickle cell anemia cohort study. PLoS ONE [Electronic Resource]. 2013;8(11):e79923. doi: <https://dx.doi.org/10.1371/journal.pone.0079923>. PubMed PMID: 24224021.

21. DeBaun MR, Rodeghier M, Cohen R, Kirkham FJ, Rosen CL, Roberts I, et al. Factors predicting future ACS episodes in children with sickle cell anemia. Am J Hematol. 2014;89(11):E212-7. doi: <https://dx.doi.org/10.1002/ajh.23819>. PubMed PMID: 25088663.

22. Garadah T, Mandeel F, Jaradat A, Bin Thani K. The effects of hydroxyurea therapy on the six-minute walk distance in patients with adult sickle cell anemia: an echocardiographic study. J Blood Med. 2019;10:443-52. doi: <https://dx.doi.org/10.2147/JBM.S203828>. PubMed PMID: 31920416.

23. Rezende PVS, M. V. Campos, G. F. Vieira, L. L. M. Souza, M. B. Belisario, A. R. Silva, C. M. Viana, M. B. Clinical and hematological profile in a newborn cohort with hemoglobin SC. J Pediatr (Rio J). 2018;94(6):666-72. doi: <https://dx.doi.org/10.1016/j.jped.2017.09.010>. PubMed PMID: 29195085.

24. Schuchard SB, Lissick JR, Nickel A, Watson D, Moquist KL, Blaylark RM, et al. Hydroxyurea use in young infants with sickle cell disease. Pediatr Blood Cancer. 2019;66(7):e27650. doi: <https://dx.doi.org/10.1002/pbc.27650>. PubMed PMID: 30729675.

25. Shome DK, Al Ajmi A, Radhi AA, Mansoor EJ, Majed KS. The effect of hydroxyurea therapy in Bahraini sickle cell disease patients. Indian J Hematol Blood Transfus. 2016;32(1):104-9. doi: <https://dx.doi.org/10.1007/s12288-015-0529-y>. PubMed PMID: 26855516.

26. van Tuijn CFJ, Schimmel M, van Beers EJ, Nur E, Biemond BJ. Prospective evaluation of chronic organ damage in adult sickle cell patients: a seven-year follow-up study. Am J Hematol. 2017;92(10):E584-E90. doi: <https://dx.doi.org/10.1002/ajh.24855>. PubMed PMID: 28699283.

27. Willen SM, Cohen R, Rodeghier M, Kirkham F, Redline SS, Rosen C, et al. Age is a predictor of a small decrease in lung function in children with sickle cell anemia. Am J Hematol. 2018;93(3):408-15. doi: <https://dx.doi.org/10.1002/ajh.25003>. PubMed PMID: 29226507.

28. Yates AM, Dedeken L, Smeltzer MP, Lebensburger JD, Wang WC, Robitaille N. Hydroxyurea treatment of children with hemoglobin SC disease. Pediatr Blood Cancer. 2013;60(2):323-5. doi: <https://dx.doi.org/10.1002/pbc.24283>. PubMed PMID: 22949140.

29. Barner J, Kang H, Richards K, Bhor M, Paulose J, Kutlar A, editors. Association between hydroxyurea adherence and persistence and vaso-occlusive crises among Texas Medicaid recipients with sickle cell disease [abstract]. Academy of Managed Care Pharmacy Managed Care & Specialty Pharmacy Annual Meeting 2019; San Diego, CA, USA; March 25-28, 2019.2019.

30. Leleu H, Arlet J, Habibi A, Etienne-Julan M, Pita M, Granghaud A, et al., editors. Epidemiology and disease burden of SCD in France: a descriptive study based on a French nationwide claims database [abstract]. Presented at: 26th Congress of the European Hematology Association; Virtual; June 20202020.

31. Aloni MN, Nkee L. Challenge of managing sickle cell disease in a pediatric population living in Kinshasa, Democratic Republic of Congo: a sickle cell center experience. Hemoglobin. 2014;38(3):196-200. doi: <https://dx.doi.org/10.3109/03630269.2014.896810>. PubMed PMID: 24669956.

32. Alsultan A, Aleem A, Ghabbour H, AlGahtani FH, Al-Shehri A, Osman ME, et al. Sickle cell disease subphenotypes in patients from Southwestern Province of Saudi Arabia. J Pediatr Hematol Oncol. 2012;34(2):79-84. doi: <https://dx.doi.org/10.1097/MPH.0b013e3182422844>. PubMed PMID: 22322941.

33. Darbari D, Onyekwere O, Nouraie M, Minniti C, Luchtman-Jones L, Rana S, et al. Markers of severe vaso-occlusive painful episode frequency in children and adolescents with sickle cell anemia. J Pediatr. 2012;160(2):286-90. doi: <https://dx.doi.org/10.1016/j.jpeds.2011.07.018>. PubMed PMID: 21890147.

34. Elmariah H, Garrett M, De Castro L, Jonassaint J, Ataga K, Eckman J, et al. Factors associated with survival in a contemporary adult sickle cell disease cohort. Am J Hematol. 2014;89(5):530-5. doi: <https://dx.doi.org/10.1002/ajh.23683>. PubMed PMID: 24478166.

35. Nimgaonkar V, Krishnamurti L, Prabhakar H, Menon N. Comprehensive integrated care for patients with sickle cell disease in a remote aboriginal tribal population in southern India. Pediatr Blood Cancer. 2014;61(4):702-5. doi: <https://dx.doi.org/10.1002/pbc.24723>. PubMed PMID: 24347362.

36. Rizio A, Bhor M, Lin X, McCausland KL, White MK, Paulose J, et al. The relationship between frequency and severity of vaso-occlusive crises and health-related quality of life and work productivity in adults with sickle cell disease. Qual Life Res. 2020;29(6):1533-47. doi: <https://dx.doi.org/10.1007/s11136-019-02412-5>. PubMed PMID: 31933113.

37. Shah N, Bhor M, Xie L, Halloway R, Arcona S, Paulose J, et al. Treatment patterns and economic burden of sickle-cell disease patients prescribed hydroxyurea: a retrospective claims-based study. Health Qual Life Outcomes. 2019;17(1):155. doi: <https://dx.doi.org/10.1186/s12955-019-1225-7>. PubMed PMID: 31619251.

38. Bronte-Hall L, Parkin M, Green C, Tchouambou D, Huynh L, Puri-Sharma C, et al., editors. Real-world clinical burden of sickle cell disease in the US community-practice setting: a single-center experience from the foundation for sickle cell disease research [abstract]. Presented at: 14th Annual Sickle Cell Disease Research & Educational Symposium; Virtual; September 22-25, 2020.2020.

39. Delicou S, Diamantidis M, Manganas K, Eftychiadis E, Pantelidou D, Kourakli A, et al., editors. Sickle-cell disease in Greece: patient reported outcomes related to clinical complications, treatment choices and attitudes, beliefs and trends affecting potential participation in clinical trials - A Greek national multicentric study [abstract]. Presented at: 61st American Society of Hematology Annual Meeting and Exposition; Orlando, FL, USA; December 7-10, 2019.2019.

40. Inati A, Al Alam C, El Ojaimi C, Hamad T, Kanakamedala H, Pilipovic V, et al., editors. Sickle cell disease burden in North Lebanon [abstract]. Presented at: 61st American Society of Hematology Annual Meeting and Exposition; Orlando, FL, USA; December 7-10, 2019.2019.

41. James J, Andemariam B, Inusa B, El-Rassi F, Francis-Gibson B, Nero A, et al., editors. Management strategies and satisfaction levels in patients with sickle cell disease: interim results from the international sickle cell world assessment survey (SWAY) [abstract]. Presented at: 61st American Society of Hematology Annual Meeting and Exposition; Orlando, FL, USA; December 7-10, 2019.2019.

42. Andemariam B, James J, Inusa B, El Rassi F, Francis-Gibson B, Nero A, et al., editors. Management strategies and satisfaction levels in patients with sickle cell disease in the US: interim results from the sickle cell world assessment survey (SWAY) [abstract]. Presented at: The Foundation for Sickle Cell Disease Research Congress; Virtual; June 2020.2020.

43. Boyd JH, Macklin EA, Strunk RC, DeBaun MR. Asthma is associated with acute chest syndrome and pain in children with sickle cell anemia. Blood. 2006;108(9):2923-7. PubMed PMID: 16690969.

44. Brousse V, El Hoss S, Bouazza N, Arnaud C, Bernaudin F, Pellegrino B, et al. Prognostic factors of disease severity in infants with sickle cell anemia: a comprehensive longitudinal cohort study. Am J Hematol. 2018;93(11):1411-9. doi: <https://dx.doi.org/10.1002/ajh.25260>. PubMed PMID: 30132969.

45. Ceglie G, Di Mauro M, Tarissi De Jacobis I, de Gennaro F, Quaranta M, Baronci C, et al. Gender-related differences in sickle cell disease in a pediatric cohort: a single-center retrospective study. Front. 2019;6:140. doi: <https://dx.doi.org/10.3389/fmolb.2019.00140>. PubMed PMID: 31867340.

46. Dave K, Chinnakali P, Thekkur P, Desai S, Vora C, Desai G. Attrition from care and clinical outcomes in a cohort of sickle cell disease patients in a tribal area of Western India. Trop. 2019;4(4):01. doi: <https://dx.doi.org/10.3390/tropicalmed4040125>. PubMed PMID: 31581481.

47. El Hoss S, Cochet S, Marin M, Lapoumeroulie C, Dussiot M, Bouazza N, et al. Insights into determinants of spleen injury in sickle cell anemia. Blood Adv. 2019;3(15):2328-36. doi: <https://dx.doi.org/10.1182/bloodadvances.2019000106>. PubMed PMID: 31391165.

48. Jaiyesimi O, Kasem M. Acute chest syndrome in Omani children with sickle cell disease: epidemiology and clinical profile. Ann Trop Paediatr. 2007;27(3):193-9. PubMed PMID: 17716447.

49. Lionnet F, Hammoudi N, Stojanovic K, Avellino V, Grateau G, Girot R, et al. Hemoglobin sickle cell disease complications: a clinical study of 179 cases. Haematologica. 2012;97(8):1136-41. doi: <https://dx.doi.org/10.3324/haematol.2011.055202>. PubMed PMID: 22315500.

50. Madu AJ, Ubesie A, Ocheni S, Chinawa J, Madu KA, Ibegbulam OG, et al. Priapism in homozygous sickle cell patients: important clinical and laboratory associations. Med Princ Pract. 2014;23(3):259-63. doi: <https://dx.doi.org/10.1159/000360608>. PubMed PMID: 24685837.

51. McClish DK, Levenson JL, Penberthy LT, Roseff SD, Bovbjerg VE, Roberts JD, et al. Gender differences in pain and healthcare utilization for adult sickle cell patients: the PiSCES project. J Womens Health. 2006;15(2):146-54. PubMed PMID: 16536678.

52. Upadhye DS, Jain DL, Trivedi YL, Nadkarni AH, Ghosh K, Colah RB. Neonatal screening and the clinical outcome in children with sickle cell disease in Central India. PLoS ONE [Electronic Resource]. 2016;11(1):e0147081. doi: <https://dx.doi.org/10.1371/journal.pone.0147081>. PubMed PMID: 26785407.
